# Supercontinuum in integrated photonics: generation, applications, challenges, and perspectives

**DOI:** 10.1515/nanoph-2022-0749

**Published:** 2023-03-01

**Authors:** Camille-Sophie Brès, Alberto Della Torre, Davide Grassani, Victor Brasch, Christian Grillet, Christelle Monat

**Affiliations:** Photonic Systems Laboratory (PHOSL), Ecole Polytechnique Fédérale de Lausanne, 1015 Lausanne, Switzerland; Université de Lyon, Institut des Nanotechnologies de Lyon (INL) UMR CNRS 5270, Ecole Centrale de Lyon, 69131 Ecully, France; Centre Suisse d’Electronique et de Microtechnique (CSEM), 2000 Neuchâtel, Switzerland; Q.ANT GmbH, 70565 Stuttgart, Germany

**Keywords:** frequency combs, integrated photonics, nonlinear optics, supercontinuum generation, waveguides

## Abstract

Frequency conversion in nonlinear materials is an extremely useful solution to the generation of new optical frequencies. Often, it is the only viable solution to realize light sources highly relevant for applications in science and industry. In particular, supercontinuum generation in waveguides, defined as the extreme spectral broadening of an input pulsed laser light, is a powerful technique to bridge distant spectral regions based on single-pass geometry, without requiring additional seed lasers or temporal synchronization. Owing to the influence of dispersion on the nonlinear broadening physics, supercontinuum generation had its breakthrough with the advent of photonic crystal fibers, which permitted an advanced control of light confinement, thereby greatly improving our understanding of the underlying phenomena responsible for supercontinuum generation. More recently, maturing in fabrication of photonic integrated waveguides has resulted in access to supercontinuum generation platforms benefiting from precise lithographic control of dispersion, high yield, compact footprint, and improved power consumption. This Review aims to present a comprehensive overview of supercontinuum generation in chip-based platforms, from underlying physics mechanisms up to the most recent and significant demonstrations. The diversity of integrated material platforms, as well as specific features of waveguides, is opening new opportunities, as will be discussed here.

## 1 Introduction

Nonlinear optics encompasses a wide range of phenomena that occur following the interaction of intense light with a material. Since the first experimental observation of one of those phenomena, second harmonic generation, by Franken et al. in 1961 [1], the interest in the fundamentals of nonlinear optics, as well as its practical implementation for the development of novel technologies, have steadily grown. Such constant evolution and flourishing of the field primarily stems from the very rich physics and our improved understanding of it. Most importantly, it also builds on the maturing of available light sources, material platforms, and nanofabrication technologies, and on the increasing computing power for simulations. This combination continuously opens up new playgrounds and opportunities for pushing the performance, control, understanding, and applications of nonlinear optical effects.

The modification of the material properties in the presence of a light field, namely the induced nonlinear polarization, not only leads to many striking phenomena, but also enables ultrafast versatile light manipulation as well as generation of light in unconventional spectral windows through nonlinear frequency conversion or extreme spectral broadening. Supercontinuum generation (SCG), the process through which a relatively intense input laser pulse undergoes significant spectral broadening while maintaining spatial coherence and high brightness, has been investigated in a wide variety of nonlinear media and has found applications such as imaging, optical coherence tomography, spectroscopy, or optical frequency comb technologies. While SCG is a relatively easy phenomenon to observe given the availability of a pulsed light source with high peak power, the control of the spectral broadening strongly depends on the linear and nonlinear properties of the medium. In that context, waveguiding systems have revolutionized SCG not only by offering enhanced light field confinement along relatively long propagation distance compared to bulk systems, but also the possibility to tailor the dispersion as to enhance and control the nonlinear interactions. State-of-the-art and commercially available supercontinuum (SC) sources are mostly based on silica glass photonic crystal fibers (PCFs), while fluoride-fiber-based middle infrared (MIR) SC sources have also become available [2–4].

In the early days of SCG, the dispersion engineering capabilities of PCFs allowed for the demonstration of SCG with enhanced performance compared to SC from bulk or conventional silica fibers. Extensive research is still pursued in fiber-based SCG and the broadest spectra are currently obtained in highly nonlinear chalcogenide fibers [5, 6]. Integrated waveguides, with the promise to further enhance light field confinement for more efficient devices, to lower power consumption accordingly and to potentially enable dense integration with additional functionalities across compact chip-based platforms, quickly appeared as a powerful and attractive approach for SCG. The recent developments in fabrication technology have finally made integrated SC sources available. The many successful recent demonstrations, reviewed here, highlight the high potential of this technology.

**Figure 1:**
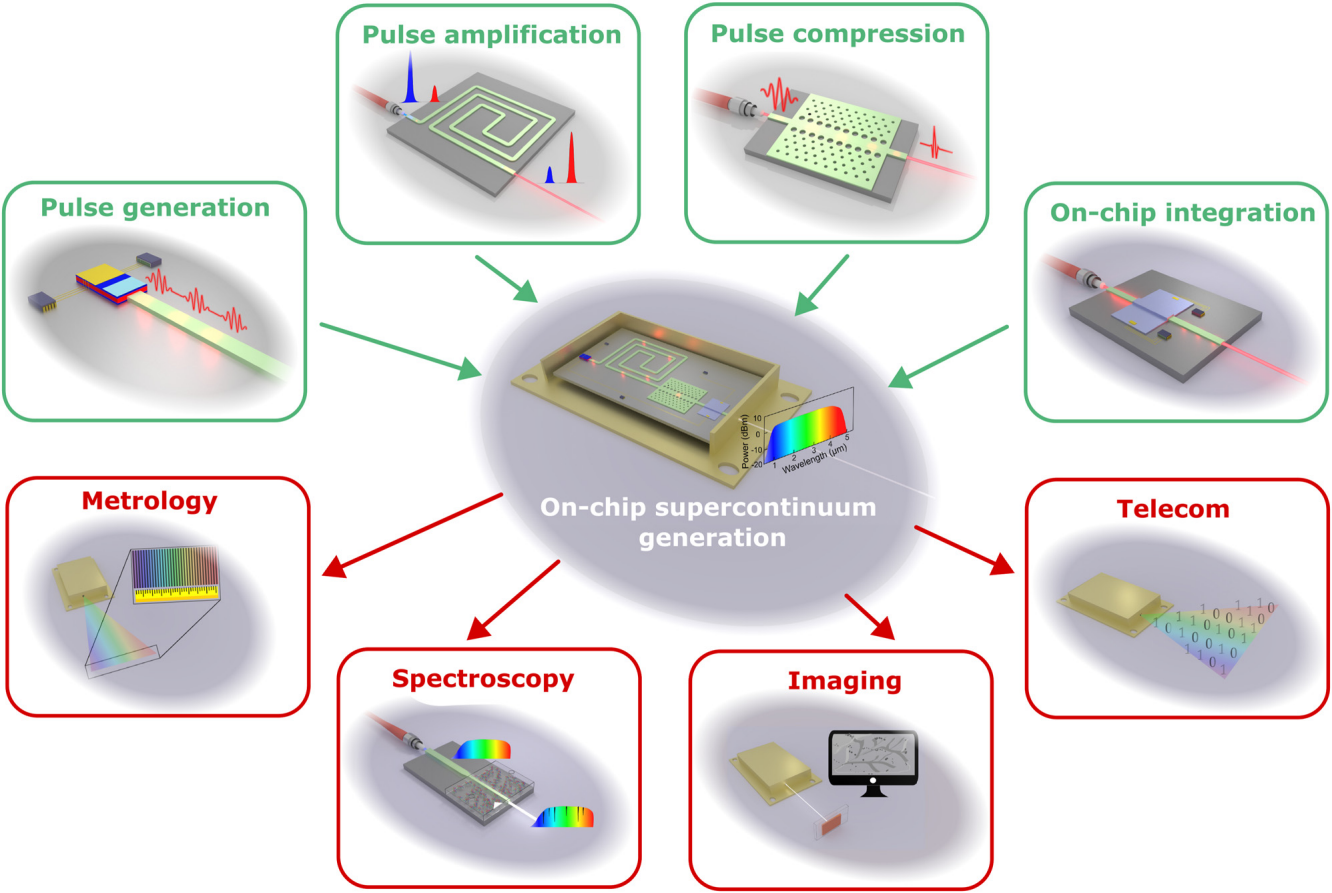
Necessary building blocks (top, green frames) for full integration of SC sources (center) and potential applications (bottom, red frames).

In this Review, we aim to not only cover state-of-the-art results in integrated SCG, but also highlight the strengths and weaknesses of the various platforms while presenting a vision for future development, as illustrated in Figure 1. The quest to achieve fully integrated SC sources requires the co-development of several building blocks such as on-chip lasers, to eliminate currently external OPAs or fiber-based lasers, as well as on-chip pulse amplification and compression to reach the required pump pulse characteristics for efficient spectral broadening. Heterogeneous integration of materials with distinct and complementary properties could also be a valuable tool. Different materials could be used at different stages along the propagation for pulse amplification and compression, to fill different spectral regions and/or tailor the waveguide properties after fabrication for more flexibility. Such developments would open up exciting and unique opportunities for a number of applications. As we will see in this Review, the potential of SC sources for metrology, spectroscopy, imaging, and high bit-rate telecommunication has been already demonstrated, although with an external pump. Other types of applications could also be envisioned, like non-invasive medical diagnosis or calibration of astronomical spectrographs for exoplanets search.

The Review is organized as follows. We start by giving an overview of the theoretical background behind the process of SCG, and highlight the important system parameters. We then focus on the use of integrated photonics for SCG, giving a description and properties of the diverse material platforms such as those based on silicon photonics, chalcogenide, and materials possessing both second (*χ* ^(2)^) and third-order (*χ* ^(3)^) nonlinearities. In the second part, we present some experimental benchmark demonstrations, highlighting the progress, optimization, and state-of-the-art results that have been obtained thus far. The main achievements are summarized in each chapter in the form of tables including waveguide dimensions, pump wavelength, nonlinearity, linear and coupling losses, coupled (peak and average) pump power, and achieved SC bandwidth. In the third part of the Review, we present applications of integrated SC sources. Finally, we discuss fundamental open questions and how to improve performances and functionalities in order to move such sources from laboratory environments into products that can eventually serve end-users.

## 2 Physics of SCG in waveguides

SCG occurs upon the propagation of a relatively short optical pulse in a nonlinear medium that can exhibit both quadratic and cubic nonlinearities, meaning that the total nonlinear polarization developed by the material is written as:

(1) $$\begin{array}{ll} P_{\text{NL}}\left(r,t\right) & =P_{\text{NL}}^{\left(2\right)}\left(r,t\right)+P_{\text{NL}}^{\left(3\right)}\left(r,t\right)\\ & =\epsilon_{0}\chi^{\left(2\right)}:EE+\epsilon_{0}\chi^{\left(3\right)}\vdots EEE \end{array}$$

*χ* ^(2)^ and *χ* ^(3)^ being the material second and third-order susceptibility, respectively. In Eq. (1) we have included just the instantaneous electronic response, and we have neglected the slower contributions from nuclei and molecular motion. Considering the envelope-based approach and a propagation in the *z* direction of the waveguide structure, the electric field **E** is expressed in the form:

(2) $$E\left(r,t\right)=Re\left[A\left(z,t\right)F\left(x,y\right)\exp\left(-\text{i}\omega_{0}t+\text{i}\beta z\right)\right]$$

where *A*(*z*, *t*) is the temporal envelope of the pulse and **F**(*x*, *y*) is the transverse modal distribution. The carrier wave has a propagation constant *β* and angular frequency *ω*_0_. Under the slowly varying envelope approximation, the pulse propagation can be modeled using a reduced scalar nonlinear envelope equation with both quadratic and cubic nonlinearities [7, 8]:

(3) $$\begin{array}{ll} & \frac{\partial A\left(z,t\right)}{\partial z}-\underset{n\geq 1}{\sum }\frac{i^{n+1}}{n!}\beta_{n}\left(\frac{\partial^{n}A\left(z,t\right)}{\partial t^{n}}\right)+\frac{\alpha }{2}A\left(z,t\right)\\ & =i\frac{\omega_{0}\overset{\infty }{\underset{-\infty }{\iint }}P_{\text{NL}}\left(z,t\right)F^{*}\left(x,y\right)\text{d}x\text{d}y}{2n_{0}c\epsilon_{0}\overset{\infty }{\underset{-\infty }{\iint }}|F\left(x,y\right){|}^{2}\text{d}x\text{d}y}. \end{array}$$

On the left hand side of the equality, the dispersion coefficients *β*_*n*_ are defined by expanding *β* as a Taylor series around *ω*_0_ and *α* is the linear loss coefficient.

When considering solely cubic nonlinearities, as it is the case for centro-symmetric and isotropic media, we can rewrite the nonlinear polarization as **P**_NL_ ≈ 3/4*ϵ*_0_ *χ* ^(3)^|*A*(*z*)|^2^ *A*(*z*)|**F**(*x*, *y*)|^2^ **F**(*x*, *y*), where we neglected the term oscillating at 3*ω*_0_ that is responsible for third harmonic generation (THG), as it is usually phase mismatched at the fundamental mode considered here (see Subsection 2.2). The nonlinear Schrödinger equation (NLSE) shown in Eq. (4), written in a reference frame moving at the pulse group velocity *v*_g_ = 1/*β*_1_, is most routinely used [9]. It should be noted that while the physical validity of the field decomposition into an envelope and a carrier can be questioned as the light pulse bandwidth approaches the carrier frequency, studies have shown that the envelope-based propagation equation can still accurately describe large spectral broadening such as in SCG.

(4) $$\begin{array}{ll} & \frac{\partial A\left(z,t\right)}{\partial z}-\underset{n\geq 2}{\sum }\frac{i^{n+1}}{n!}\beta_{n}\left(\frac{\partial^{n}A\left(z,t\right)}{\partial t^{n}}\right)+\frac{\alpha }{2}A\left(z,t\right)\\ & \;=i\gamma |A\left(z,t\right){|}^{2}A\left(z\right), \end{array}$$

where

(5) $$\gamma =\frac{3\chi^{\left(3\right)}\omega_{0}}{8n_{0}c}\frac{\overset{\infty }{\underset{-\infty }{\iint }}|F\left(x,y\right){|}^{4}\text{d}x\text{d}y}{\overset{\infty }{\underset{-\infty }{\iint }}|F\left(x,y\right){|}^{2}\text{d}x\text{d}y}$$

is the waveguide nonlinear coefficient. Although in Eq. (2) the field’s amplitude *A*(*z*, *t*) has units (V/m), it is often practical to normalize it such that |*A*(*z*, *t*)|^2^ corresponds to the total transmitted power *P*, which is expressed in Watt and can be obtained by integrating the Poynting vector over the cross-section of the waveguide: $P=\frac{\epsilon_{0}n_{0}c}{2}|A\left(z\right){|}^{2}\iint_{-\infty }^{\infty }|F\left(x,y\right){|}^{2}$ . In this way, we can define the effective area of the waveguide mode underlying the pulse propagation, which is typically the fundamental mode, as

(6) $$A_{\text{eff}}=\frac{\overset{\infty }{\underset{-\infty }{\iint }}|F\left(x,y\right){|}^{4}\text{d}x\text{d}y}{{\left(\overset{\infty }{\underset{-\infty }{\iint }}|F\left(x,y\right){|}^{2}\text{d}x\text{d}y\right)}^{2}}.$$

Also, the nonlinear coefficient can be rewritten in terms of the nonlinear refractive index *n*_2_ as *γ* = *ω*_0_ *n*_2_/(*cA*_eff_), with *n*_2_ being related to the diagonal element of the *χ* ^(3)^ tensor through $n_{2}=\frac{3\chi^{\left(3\right)}}{4\epsilon_{0}cn_{0}^{2}}$ . It is also possible to include nonlinear absorption and associated free-carrier absorption depending on the medium and pumping scheme used. Eventually, Eq. (4) can be rewritten to include the delayed Raman contribution [9] as:

(7) $$\begin{array}{ll} & \frac{\partial A\left(z,t\right)}{\partial z}-\underset{n\geq 2}{\sum }\frac{i^{n+1}}{n!}\beta_{n}\left(\frac{\partial^{n}A\left(z,t\right)}{\partial t^{n}}\right)+\frac{\alpha }{2}A\left(z,t\right)\\ & \;=i\gamma \left(1+\frac{i}{\omega_{0}}\frac{\partial }{\partial t}\right)\\ & \;\;\times \left(A\left(z,t\right)\overset{\infty }{\underset{-\infty }{\int }}R\left(t^{\prime }\right)|A\left(z\right.,t-t^{\prime }{|}^{2}dt^{\prime }\right), \end{array}$$

with $R\left(t\right)=\left(1-f_{r}\right)\delta \left(t\right)+f_{r}h_{r}\left(t\right)$ , *f*_*r*_ the fractional contribution and *h*_*r*_(*t*) the temporal response.

**Figure 2:**
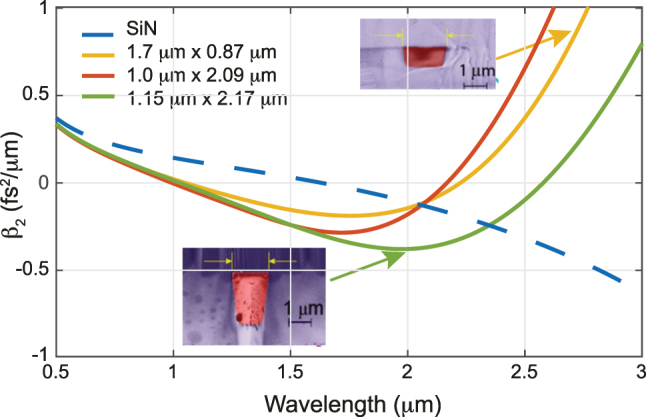
Group velocity dispersion (*β*_2_) for the TE fundamental mode of a standard silicon nitride waveguide with width × height cross-section 1.7 μm × 0.87 μm and the TM fundamental mode of 1.0 μm × 2.09 μm and 1.15 μm × 2.17 μm waveguides with dimension. The material GVD of SiN (in its stoichiometric form) is shown as a dashed line. The insets show the scanning electron microscope (SEM) images of the waveguide cross-sections.

The dispersion and its interplay with the nonlinearities strongly influence the spectral broadening as different physical phenomena can occur. The dispersion of a propagating mode results from material, waveguide, modal, and polarization contributions. The latter two only come into play for multi-mode transmission or transmission of different polarizations, and can be, for most SCG, left aside. The material dispersion comes from the frequency dependence of the refractive index of the bulk material. For a given material platform (composed of distinct core and cladding materials), the mode dispersion can be further modified and controlled by the waveguide geometry, thereby potentially counteracting the effect of material dispersion. Such waveguide dispersion engineering allows obtaining a specific dispersion profile as illustrated in Figure 2, where the second order dispersion coefficient *β*_2_, also called group velocity dispersion (GVD), of silicon nitride (SiN) waveguides for different waveguide core height and width are plotted. The dispersion parameter *D* = −2*πc*/*λ* ^2^ × *β*_2_ is also often used as an alternative metric to the GVD. Such dispersion engineering is crucial to control the underlying dynamics of SCG. In particular, the GVD describes the relative group velocity of the spectral components of the pulse. Practically, the GVD induces a phase delay on each frequency component with respect to that of the center frequency, leading to temporal pulse broadening. The characteristic length of second-order dispersion is given by $L_{\text{D}}=T_{0}^{2}/|\beta_{2}|$ and corresponds to the length after which an initially unchirped Gaussian pulse with a 1/*e* half intensity width of *T*_0_ will have broadened by $\sqrt{2}$ . Depending on the sign of the GVD, two different regimes can occur. In the normal dispersion regime, when *β*_2_ > 0 (or *D* < 0), high frequency components are slowed down compared to low frequency ones. The opposite occurs in the anomalous dispersion regime (*β*_2_ < 0 or *D* > 0). Waveguide dispersion engineering allows to shift the boundaries between the normal/anomalous regions (which occur at zero dispersion wavelengths – ZDW), to counterbalance material dispersion, and, to a certain extent, to control the value of dispersion so as to lower it or flatten it in a targeted spectral region, as seen in Figure 2. It can be noted that, compared to PCFs, the *β*_2_ values for waveguides are typically larger and that, owing to the larger core/cladding index contrast, slight changes in the waveguide cross-section induce a larger change in dispersion. While this advantageously allows for high tunability of the dispersion curve, it remains constrained by the fabrication tolerances and maturity.

The main driving nonlinear effect contributing to SCG early during the propagation is self-phase modulation (SPM), which results from the intensity (*I*) dependent refractive index also known as the optical Kerr effect: *n*(*ω*, *I*) = *n*_0_(*ω*) + *n*_2_ *I*. SPM is characterized, in the frequency domain, by the appearance of sidelobes around the central frequency. The accumulated frequency-dependent phase leads to a frequency chirp imposed on the pulse with red-shifted components at the leading edge of the pulse and blue-shifted ones at the trailing edge. Similar to dispersion, a characteristic length of SPM (i.e. nonlinearity) is defined by the following expression *L*_NL_ = 1/(*γP*_0_). The nonlinear length *L*_NL_ corresponds to the propagation length required for a pulse of input peak power *P*_0_ to accumulate 1 rad of nonlinear phase shift in a waveguide with a nonlinear coefficient *γ*. As will be shown below, the interaction between GVD and SPM can lead to different SCG regimes, with both distinct dynamics and resulting spectral and temporal properties. Besides this interplay, another driving mechanism called modulation instability (MI) can strongly affect the dynamics and characteristics of the SC. In the anomalous dispersion regime, small perturbations in the form of residual noise signal spectrally detuned from the pump, can be amplified through phase-matched four-wave mixing processes resulting in MI. As a noise seeded and highly stochastic process [10], MI leads to significant phase and intensity fluctuations which have an influence in the coherence property of the SCG. We briefly describe, in the next two sections, the SCG associated with the two distinct dispersion regimes, and discuss, respectively, the resulting SC characteristics.

### 2.1 SCG in anomalous dispersion regime

A large majority of work on SCG relies on pumping in the anomalous dispersion, since SC can then be readily generated by leveraging the opposite effects of SPM and GVD that govern the generation of solitons and their dynamics. Optical solitons are self-stabilized wave packets that arise from the delicate balance between nonlinear and dispersive effects. Physically, the soliton regime requires that *L*_D_ ≥ *L*_NL_ and the pulses are then characterized by a soliton number *N* given by:

(8) $$N^{2}=\frac{L_{\text{D}}}{L_{\text{NL}}}=\frac{\gamma P_{0}T_{0}^{2}}{\left|\beta_{2}\right|}$$

Fundamental solitons with undistorted pulse spectrum and temporal envelope arise when *L*_D_ = *L*_NL_. For higher-order solitons, as typically involved in SCG, the initial spectral broadening caused by SPM and subsequent temporal compression are proportional to the soliton number. At the compression point, when the spectrum is dramatically broadened, the soliton of order *N* is generally perturbed, leading to soliton fission such that the resulting *N* fundamental solitons split in time and all-together cover a much broader spectrum. The characteristic length at which compression occurs is often given by:

(9) $$L_{\text{fiss}}=\sqrt{L_{\text{D}}L_{\text{NL}}}=\frac{L_{\text{D}}}{N}=\frac{T_{0}}{\sqrt{\gamma P_{0}|\beta_{2}|}}$$

The main perturbing mechanisms causing soliton fission are higher-order dispersion (HOD) and nonlinear terms such as stimulated Raman scattering. When the broadened spectrum of the optical pulse overlaps the normal dispersion regions, the soliton pulses will shed energy to resonant dispersive waves (DW) spectrally shifted from the pump on the other side of the ZDW(s), in a phenomenon analogous to the emission of Cherenkov radiation. Typically, only the main high power soliton created after the fission can lead to the generation of DW. Efficient energy transfer occurs at the frequency where the phase constant of the main soliton pulse at frequency *ω*_s_ and peak power *P*_s_ equals the one of the linear DW. This is given by the phase-matching condition:

(10) $$\beta \left(\omega \right)-\beta \left(\omega_{\text{s}}\right)-v_{\text{g}}^{-1}\left(\omega -\omega_{\text{s}}\right)-\frac{\gamma P_{\text{s}}}{2}=0$$

where the soliton has a group velocity *v*_g_. Neglecting the nonlinear contribution $\left(\frac{\gamma P_{\text{s}}}{2}\right)$ , the left-hand side of Eq. (10) is called integrated dispersion *β*_int_, and can be rewritten as a Taylor expansion:

(11) $$\beta_{\text{int}}\left(\omega \right)=\underset{k\geq 2}{\sum }\frac{{\left(\omega -\omega_{\text{s}}\right)}^{k}}{k!}\frac{\partial^{k}\beta }{\partial \omega^{k}}$$

**Figure 3:**
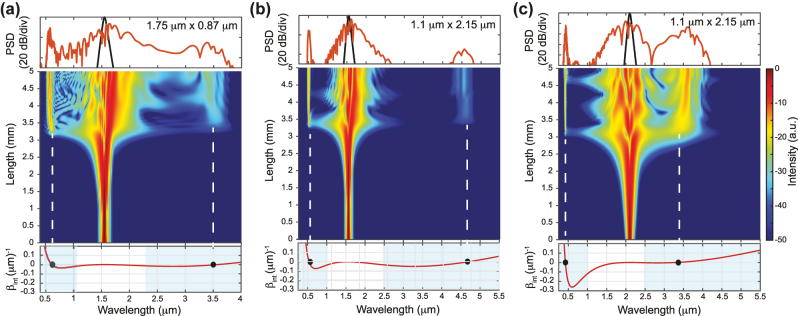
Illustration of dispersive wave engineering. Simulated spectrum on top (black – input, red – output), simulated spectral evolution along propagation in the middle and calculated integrated dispersion at the bottom for: (a) 1.75 μm × 0.87 μm waveguide pumped at 1550 nm in TE; (b) 1.1 μm × 2.15 μm waveguide pumped at 1550 nm in TM; (c) 1.1 μm × 2.15 μm waveguide pumped at 2100 nm in TM. The positions of the expected DWs are indicated by black dots in the integrated dispersion (*β*_int_ = 0) and align well with the spectrum simulation highlighted by the white dashed lines. The regions of normal dispersion are identified in light blue in the bottom graphs. Numerical simulations are performed by solving the GNLSE for the same peak pump power of 5 kW.

The GVD determines the location of the phase matching points, and it has been shown that the generation of a phase matched DW corresponds to the occurrence of a ZDW, as the DW will be generated in the normal dispersion regions. The engineering of waveguide dispersion, and thus control of the waveguide geometry, allows designing SC sources with DWs generated on both the short and long wavelength side of the pump when the latter is positioned between two ZDWs. Leveraging the generation of DWs allows for a significant increase of the SC bandwidth. Additionally, HOD terms (*k* > 2) affect the amount of power transfer to the DW: even-order dispersion terms lead to two DWs with symmetric intensity and frequency detuning with respect to the pump, while positive or negative odd-order terms break this symmetry favoring blue or red-shifted DW, respectively [11]. Simulations of different scenarios are shown in Figure 3 to highlight the importance of DW engineering. In this figure, the calculated *β*_int_ and simulated spectral evolution along a 5 mm length together with the final output spectrum are shown for 2 different waveguide geometries (1.75 μm × 0.87 μm for Figure 3(a) and 1.1 μm × 2.15 μm for Figure 3(b) and (c)) and two different pump wavelengths (1550 nm for Figure 3(a) and (b) and 2100  nm for Figure 3(c)). One can see an excellent agreement between the expected position of the DWs provided by numerical simulations and the wavelength satisfying the phase matching condition *β*_int_ = 0 when neglecting the nonlinear contribution from the *β*_int_ calculation. It is also clear that due to varying GVD at the pump wavelength between the three scenarios, the wavelength of the DW changes but also the total amount of accumulated integrated dispersion at the red or blue detuned side. For these particular examples, while the wavelength of the red detuned DW does not significantly vary for the 1.75 μm × 0.87 μm pumped at 1550 nm (Figure 3(a)) and the 1.1 μm × 2.15 μm waveguide pumped at 2100 nm (Figure 3(c)), the accumulated dispersion towards the blue side is significantly higher for the latter. One would therefore expect, for the larger cross-section waveguide, an imbalance in the energy transfer (and in the bandwidth of the generated DW owing to the different *β*_int_ slopes) between the blue-detuned DW and the red-detuned DW compared to the smaller cross-section waveguide. Additionally, one should also consider the spectral overlap between the soliton (roughly located around the pump wavelength) and the DW to estimate the actual conversion efficiency towards the DW.

The corresponding numerical simulations support the correlation between symmetry breaking in *β*_int_ and imbalance in the power transfer. In Figure 3(a), the amount of energy transferred to the blue-detuned DW, compared to the red-detuned one located near 3.5 μm, is significantly higher since the *β*_int_ is almost symmetric around the pump wavelength but the broadened soliton has a better spectral overlap with the blue DW. Instead, for the large cross-section design, Figure 3(c), a significant improvement is observed on the long wavelength DW, also located near 3.5 μm, since it is closer to the 2.1 μm pump signal. As such the overlap with the pump broadened spectrum is improved while the asymmetry in *β*_int_ further strengthens the energy transfer towards this red DW. When the 1.1 μm × 2.15 μm waveguide is pumped at 1550 nm (Figure 3(b)), the pump is nearer the maximum value of GVD (i.e. in between the two ZDWs) and the red DW is expected to be further detuned, reaching 4.6 μm (Figure 3(b)). However, despite a relatively low accumulated dispersion on the red DW side, the large DW/pump detuning results in poor conversion efficiency to the red detuned DW, resulting in a poor MIR source.

**Figure 4:**
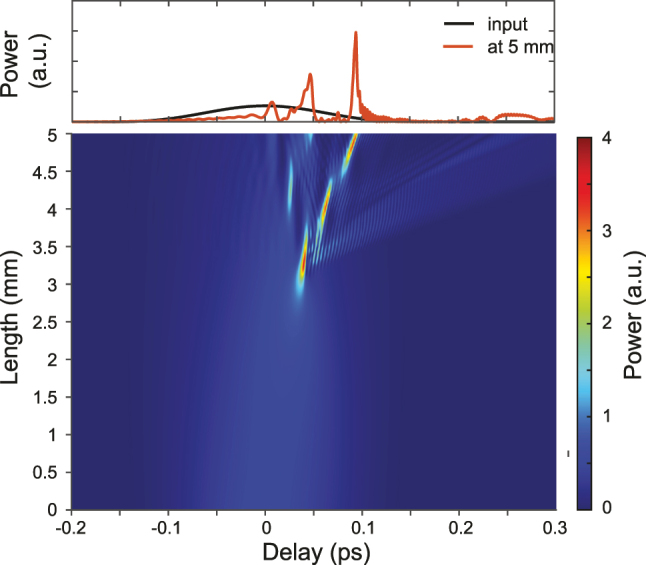
Simulated temporal envelope on top (black – input, red – output), and temporal pump pulse evolution in the bottom, corresponding to the same conditions as in Figure 3(a).

The temporal evolution corresponding to the same conditions used for the spectral evolution of Figure 3(a) is shown in Figure 4. We clearly see that after the first compression point (around 3 mm of propagation), the propagating pulse, which is significantly compressed, splits while DWs are emitted. The DWs rapidly walk off from the pump temporal residue. The stronger visible DW can be seen on the trailing edge of the temporal window owing to the increased group-delay as compared to the pump residue.

While pumping in the low anomalous dispersion allows maximizing the soliton number, and therefore pulse compression and spectral broadening, the competition of coherent and incoherent spectral broadening dynamics, namely soliton fission and MI, governs the coherence properties of the resulting spectrum. For some applications, a high degree of coherence may be required as to, for example, maintain the frequency comb nature of the pumping source over the entire SC spectrum. Indeed if the pump source is a frequency comb, characterized by its repetition rate *f*_rep_ and offset frequency *f*_CEO_, both in the microwave range, such that every comb line at optical frequency *f*_*n*_ is expressed as *f*_*n*_ = *f*_CEO_ + *nf*_rep_, coherent broadening will transfer the comb properties to the entire SC spectrum. A quick assessment of the expected coherence can be obtained through the calculated complex degree of first-order coherence $g_{12}^{\left(1\right)}$ is given by:

(12) $$g_{12}^{\left(1\right)}\left(\lambda \right)=\frac{\left\langle E_{1}^{*}\left(\lambda \right)E_{2}\left(\lambda \right)\right\rangle }{\sqrt{\left\langle |E_{1}\left(\lambda \right){|}^{2}\right\rangle \left\langle |E_{2}\left(\lambda \right){|}^{2}\right\rangle }},$$

where the angle brackets denote an ensemble average over independent supercontinuum complex spectral envelopes *E*_1_ and *E*_2_ for different noise settings. It is possible to ensure good coherence (i.e. phase stability at each wavelength across the spectrum) of anomalous SCG, the main approach being to maintain a low soliton number. Given the two competing effects, the generation of a coherent spectrum requires that soliton fission dominates, meaning that *L*_fiss_ must be significantly shorter than the distance over which MI impacts the evolution dynamics (*L*_MI_). It was estimated that *L*_MI_ ∼ 16*L*_NL_ [12] meaning that a soliton number *N* < 16 would ensure a high level of coherence. From a system’s perspective, it is therefore preferable to use waveguides only slightly longer than *L*_fiss_, and pump them with ultrashort pulses (in the fs scale) at high values of anomalous GVD. All these are possible in integrated waveguides. As will be discussed in the next section, another approach to guarantee coherence is to pump in the normal dispersion regime, since the distinct driving mechanisms underpinning SCG do naturally preserve coherence.

### 2.2 SCG in normal dispersion regime

When a pulse propagates in the normal dispersion regime, the main mechanism behind spectral broadening is SPM. Because the chirp imposed on the pulse spectrum by normal dispersion and by SPM has the same sign, the spectral broadening obtained in such regime is relatively narrow. Indeed, the dispersion-induced pulse temporal stretching is no longer compensated by the counteracting Kerr effect, as in the anomalous dispersion regime, leading to a fast decrease of the pulse peak power. However, the interaction between GVD and SPM in the normal dispersion regime gives rise to other phenomena that help to increase the spectral broadening, such as optical wave breaking (OWB). In the first stages of propagation, the major physical effect acting on a pulse is SPM which, as previously mentioned, introduces a chirp with a typical S-shape across the pulse duration. GVD leads to a further steepening at the edges of the pulse spectrum such that, after a specific distance *L*_WB_:

(13) $$L_{\text{WB}}=\frac{L_{\text{D}}}{\sqrt{4N^{2}\text{exp}\left(-3/2\right)-1}}$$

the red-shifted components near the pulse leading edge travel faster and overtake the slower preceding tail, while the opposite happens for the blue-shifted wavelengths at the trailing tail, resulting in OWB [13] and the further creation of new frequencies from beating and FWM. Interestingly, OWB leads to a significant enhancement of the spectrum flatness and a nearly linear distribution of the instantaneous frequency across the pulse duration, which is particularly interesting for pulse re-compression and lies in contrast to the pulse splitting effect observed in the anomalous dispersion regime. Finally, because the dynamics of SPM and OWB generation are much less susceptible to noise amplification than soliton dynamics, the resulting SC possesses high phase stability and coherence [14].

**Figure 5:**
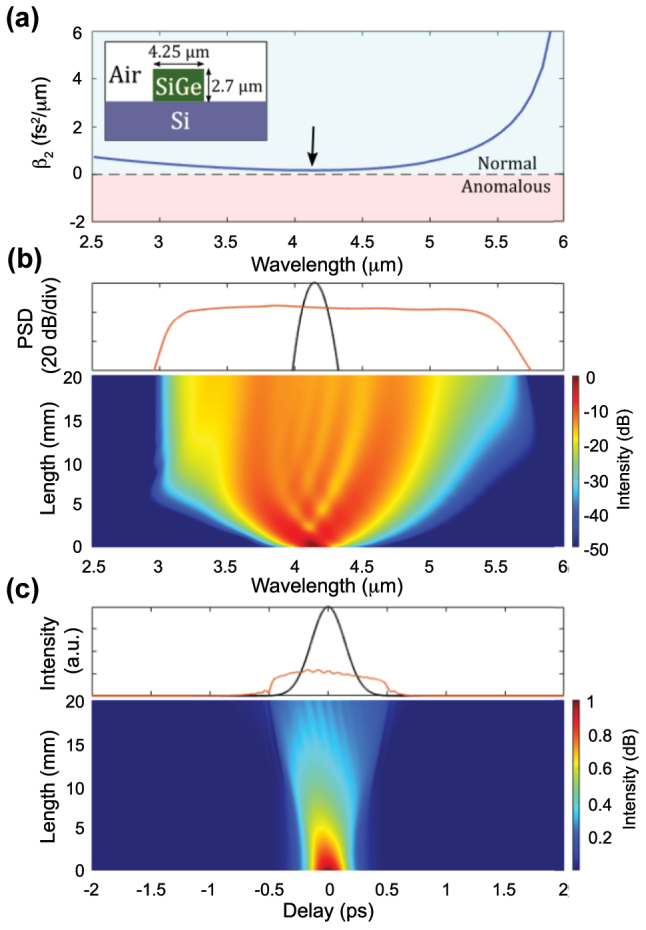
SCG in normal dispersion regime. (a) Simulated group velocity dispersion (*β*_2_) for the fundamental TM mode of a SiGe/Si waveguide. The arrow indicates the position of the pump at around 4.15 μm. Inset: schematic of the SiGe waveguide geometry. (b) Simulated normalized spectral evolution (bottom) along the propagation direction, input (black) and output (red) spectra (top) when 200 fs pulses with 3.1 kW peak power are injected in the TM mode. (c) Corresponding temporal evolution along the propagation direction (bottom), input (black) and output (red) temporal envelope (top).

An example of the generation of all-normal dispersion (ANDi) SC is shown in Figure 5 based on the waveguide used in [15]. This air clad waveguide exhibits ANDi for the TM mode (Figure 5(a)). The simulation of the spectral evolution along the waveguide propagation, shown in Figure 5(b), confirms the ANDi regime. The spectrum is flat and smooth, with an expected high coherence. The chirp is found to be quasi-linear along the pulse duration, while the pulse maintains its integrity in the time-domain (Figure 5(c)). The slight asymmetry observed between the short and long wavelength sides of the spectrum is due to the asymmetric dispersion profile. We re-emphasize that the spectral broadening of the resulting SCG tends to be significantly narrower though, and typically requires larger peak power than that needed in the anomalous dispersion regime. Apart from these two distinct SCG dynamics, as governed by the underlying waveguide dispersion sign, additional effects, such as harmonic generation discussed next, can be helpful and contribute to further broaden the SC spectrum.

### 2.3 Harmonic generation

Besides the broadening of the input spectrum, nonlinear effects can also multiply the frequency of the input light within harmonic generation (HG) processes such as second harmonic generation (SHG) and THG. These can be particularly significant in single or hybrid chip-based material platforms that exhibit both a *χ* ^(2)^ and *χ* ^(3)^ nonlinear response. HG becomes very apparent, for instance, in some nonlinear waveguides, which often start to glow green when pumped with intense pulses around 1550 nm. At the origin of this is THG (hence near 520 nm), which is driven by the same Kerr nonlinearity as that underlying many of the SCG processes described above. HG often manifests itself as the creation of light signals at frequencies that are spectrally well defined around the multiples of the pump frequency. Note that for sufficient pump power, HG conversion efficiency can be high and even occur earlier than SCG along the propagation direction. In this case, it may be difficult to distinguish the effects of HG and SCG at the harmonic frequencies, both of them interacting to eventually broaden the overall spectrum even further. Indeed, when the efficiency of the two processes is comparable, frequency mixing between different SC spectral bands or broadband HG created from the SC generated around the (fundamental) pump frequency can be observed too. As for many nonlinear mechanisms, HG processes can be enhanced by phase matching, which could be achieved here by exploiting different spatial modes in the waveguide. Whereas most of the work in the Review, and in particular the theory summarized above, assumes that the light propagates in one of the waveguide fundamental (TE or TM) modes, waveguides generally are not truly single mode at the pump wavelength. Moreover, at shorter wavelengths, around the second and third harmonic of the pump, many more modes exist for typical waveguide geometries used for SCG [16]. Some of these higher-order modes can phase-match a part of the pump spectrum propagating along the fundamental mode and therefore satisfy a condition leading to an efficient nonlinear energy transfer to these shorter frequency harmonic bands. Therefore, HG can be used to substantially and coherently expand the spectral bandwidth of SC towards shorter wavelengths, in particular for integrated platforms combining *χ* ^(2)^ and *χ* ^(2)^ nonlinearities.

One important aspect to keep in mind is that broadband spectra generated from a combination of coherent SCG and HG does not usually represent a single proper frequency comb, unless the *f*_CEO_ of the pump is 0. Instead, the fundamental SC spectrum and the HG spectra represent different frequency combs which are shifted one against the other by the *f*_CEO_, a fact that is exploited in the f-to-2f scheme that will be described in Section 7.1. However, for many applications this detail is not relevant and HG can be used to substantially expand the spectral bandwidth of SC towards shorter wavelengths.

After this description of the most common physical mechanisms underlying SCG, we will review some specific features of integrated platforms that are relevant for SCG and briefly discuss their potential for these applications, in particular with respect to their SCG fiber counterparts.

### 2.4 Nonlinear platforms for integrated SCG

Whether it be for SCG based on soliton dynamics or ANDi broadening, integration offers interesting benefits following the large achievable nonlinear coefficient *γ*. This comes from both the typically reduced effective area compared to fiber platforms but also from the wide range of integration-compatible materials with high *n*_2_ (typically 10 to 100 times higher with respect to that of silica) that can be heterogeneously integrated on the same chip-based platform. Table 1 summarizes some properties of the materials that have been most commonly used for creating chip-based SCG sources: silica (SiO_2_,) Hydex, silicon (Si), silicon nitride (SiN) in its stoichiometric composition (Si_3_N_4_), silicon germanium (SiGe), germanium (Ge), wide band-gap semiconductors such as aluminum nitride (AlN), aluminum gallium arsenide (AlGaAs), indium gallium phosphide (InGaP), chalcogenide glass (ChG), lithium niobate on insulator (LNOI), silicon carbide (SiC), and diamond.

**Table 1:** Properties of materials used for optical integration. Values of refractive index *n*, and *n*_2_, are at 1.55 μm unless stated otherwise. The transparency window is defined as the band where the absorption loss is below 2 dB/cm.

Material	Transparency window (μm)	Bandgap (*E*_g_)	*n*	*n*_2_ (cm^2^/W)	Ref.
SiO_2_	0.13–3.5	9 eV	1.46	∼2.7 × 10^−16^	[17, 18]
Hydex	0.13–3.5	9 eV	1.7	∼1.1 × 10^−15^	[19]
Si	1.1–9	1.12 eV	3.48	∼6 × 10^−14^	[20]
Si_3_N_4_	0.35–7	5 eV	2	∼2.4 × 10^−15^	[21]
SiGe (40% Ge)	1.5–11	∼1.1 eV	3.6 (at 4 μm)	∼4 × 10^−14^ (at 4 μm)	[15, 22, 23]
Ge	2–14	0.7 eV	4 (at 4 μm)	0.5–1 × 10^−13^ (in MIR)	[23–27]
AlGaAs	0.574/0.872–>6.5	1.42–2.16 eV	2.86–3.5	∼10^−13^	[28–31]
AlN	0.2–>5.5	6.2 eV	2.21(o), 2.26(e)	∼2.3 × 10^−15^	[32–35]
InGaP	0.65–MIR	1.9 eV	3.13	∼10^−13^	[36–38]
ChG	Sulphides: VIS-11	Sulphides: ∼2.5 eV	2–3	9 × 10^−12^–9 × 10^−14^	[39–41]
	Selenides: NIR-15	Selenides: ∼1.8 eV			
	Tellurides: NIR-20	Tellurides: ∼1.5 eV			
LNOI	0.35–5	∼4	2.21 (o), 2.13 (e)	∼1.8 × 10^−15^	[42–44]
SiC	0.4–MIR	2.36–3.23	2.6	∼1 × 10^−14^	[45, 46]
Diamond	0.22–MIR	5.5 eV	2.38	∼0.8 × 10^−15^	[47]
Ta_2_O_5_	0.3–8	3.8 eV	2.05	∼2 × 10^−14^	[48]
TiO_2_	0.4–5.9	3.4 eV	2.4	∼2.4 × 10^−14^	[49]
TeO_2_	0.4–5	3.8 eV	2.1	∼1.3 × 10^−14^	[50]

It is interesting to note that the typical design rules for broadband SCG in fiber platforms, which require a low GVD at the pump wavelength and long length of propagation, cannot be directly transferred to integrate waveguides, in particular the ones with high values of *γ*. The low GVD and long length requirements in fibers come from the fact that the achievable spectral broadening increases with the soliton number. For a finite pump power and a given pulse duration, a low *γ*, which is typical of fiber platforms, implies that the pump must be located at a low value of *β*_2_ (i.e. near a ZDW) according to Eq. (8). As a consequence, only one DW is typically excited even if the waveguide has 2 (distant) ZDWs. Additionally, following Eq. (9), *L*_fiss_ is relatively long, requiring in general cm to m propagation lengths. For integrated waveguides with their enhanced *γ* (several orders of magnitude larger than standard fibers), however, much higher values of GVD can be tolerated without sacrificing the soliton number. Consequently, pumping can be done near the maximum GVD value for instance, allowing for the generation of two spectrally distant and well-separated DWs, as already depicted in Figure 3. The high value of GVD also reduces the fission length, resulting in device lengths of only a few mm. Eventually, all of these considerations for integrated waveguides, leading to a reduction of the main physical length scales (*L*_D_, *L*_NL_ and *L*_fiss_) involved in SCG, open up unique opportunities to generate SC in chip-based miniaturized devices.

One way to benchmark the performance of various platforms for SCG is to use a figure of merit (FOM) that conveys the typical need to obtain the broadest SC bandwidth for as low a power-device length budget as possible. We thus define it as:

(14) $$\text{FOM}=\frac{\Delta \nu }{LP_{\text{P}}}$$

**Figure 6:**
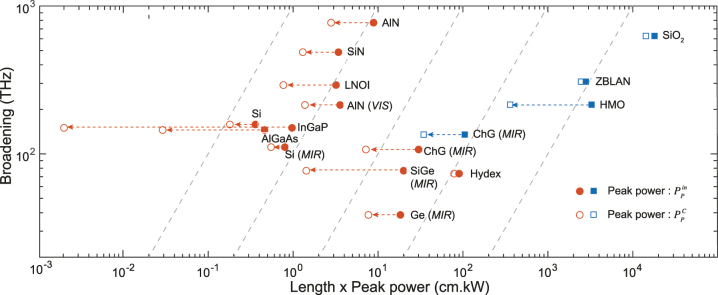
FOM and performance comparison. Spectral broadening (in THz) as a function of the product length × peak power. The squares are fiber platforms while the circles are integrated platforms. Full markers are calculated based on the estimated peak power at the input waveguide $\left(P_{\text{P}}^{\text{in}}\right)$ while the open markers are for the coupled peak power $\left(P_{\text{P}}^{\text{C}}\right)$ . If the pump wavelength is not in the near-IR (telecom band around 1550 nm), the pumping band is indicated: it can be in the near visible (VIS) around 780 nm or in the middle infrared (MIR) between 3 and 8 μm. Dashed lines are indication of constant FOM from 0.1 to 1000 (right to left).

Our FOM therefore compares the SC broadening (Δ*ν*) obtained at the output for a given pump peak power (*P*_P_) and device length (*L*). The FOM can be calculated either considering the peak power required at the input of the fiber/waveguide $\left(P_{\text{P}}^{\text{in}}\right)$ or coupled inside the fiber/waveguide $\left(P_{\text{P}}^{\text{C}}\right)$ . Since the latter does not consider the coupling losses, it represents the actual power needed to initiate the nonlinear effects in the waveguide, thereby effectively increasing the inferred FOM, but giving a better indication of the nonlinear potential of the platform for sustaining SCG. Alternatively, considering the required power at the input of the device (hence considering coupling losses), gives an indication of the overall device requirements, also reflecting the present maturity of the technology for one given material platform. Note that for waveguide-based platforms, strategies have been developed to mitigate the coupling loss issue, which tends to be more challenging than in fibers. The FOM comparison results are summarized in Table 2 (using $P_{\text{P}}^{\text{in}}$ ). Accordingly, Figure 6 shows the broadening obtained in THz as a function of the *LP*_P_ product for various experimental SCG demonstrations found in the literature, including fiber platforms (circles), made from SiO_2_ [51], heavy metal oxide glass [52], fluoride fibers (ZBLAN) [53] and chalcogenide (ChG) [54], and integrated platforms (square) from Hydex [55], Si_3_N_4_ [56], Si [57, 58], SiGe [59], Ge [25], ChG [60], AlN [33, 61], AlGaAs [28], InGaP [38], and LNOI [62]. The dashed lines represent constant FOM as guides for the eye. The full markers are obtained considering $P_{\text{P}}^{\text{in}}$ , while the open data markers are inferred for $P_{\text{P}}^{\text{C}}$ . Again, the latter show the intrinsic nonlinear potential of one given material platform for SCG after factoring out the coupling strategy deployed for the related demonstration.

**Table 2:** FOM for various material platforms as fibers (first 4 rows) or integrated waveguides and the wavelength of the pump used (λ_pump_).

Material	*λ*_pump_ (nm)	FOM (THz/(cm kW)
SiO_2_ PCF [51]	1064	3.49 × 10^−2^
Heavy metal oxyde (HMO) glass PCF [52]	1580	6.6 × 10^−2^
ZBLAN fiber [53]	1553	1.1 × 10^−1^
ChG nanowire [54]	2874	1.29 × 10^0^
Hydex [55]	1290	8.16 × 10^−1^
SiN [56]	1550	1.13 × 10^2^
Si [57]	1950	4.39 × 10^2^
Si [58]	3060	1.39 × 10^2^
SiGe [59]	8500	3.84 × 10^0^
Ge [25]	4600	2.1 × 10^0^
ChG [60]	4184	3.56 × 10^0^
AlN [61]	780	5.99 × 10^1^
AlN [33]	1560	8.71 × 10^1^
AlGaAs [28]	1555	3.15 × 10^2^
InGaP [38]	1550	1.55 × 10^2^
LNOI [62]	1550	9.13 × 10^1^

As evident from Table 2, the FOM of integrated platforms can be several orders of magnitude higher than in fiber platforms, even when the calculation is based on the peak pump power required at the input of the device. This is also seen in Figure 6. As expected, when using, alternatively, the coupled peak power to estimate the FOM, integrated platforms show an even greater performance than fibers as they are typically characterized by much higher coupling losses. This is particularly striking for less mature platforms such as GaInP, AlGaAs, SiGe, or ChG. Overall, the gain in FOM mostly comes from the significantly reduced required propagation length × pump power consumption budget afforded by chip-based platforms to trigger SCG with respect to fibers, since the obtained broadening in fiber platforms has close to record values, especially in the near-IR. We can also notice that the FOM of typical MIR integrated platforms, ChG, SiGe, and Ge, tend to be lower and closer to the FOM of fiber platforms. This can be partly explained by their waveguide design rules, which become similar to the ones of fiber based systems when operating at longer wavelengths: pumping is typically done at a wavelength of low GVD and already in the MIR, while the waveguide cross-section becomes significantly larger than for near-IR operation as to accommodate MIR light, hence leading to an overall reduced waveguide nonlinear coefficient *γ*, higher pump powers, and smaller broadening.

Overall, it becomes clear that integrated waveguides have a form factor very well adapted for SCG, enabling the creation of both compact and low power consumption sources that benefit from tight light confinement, advanced dispersion control, and integration of highly nonlinear materials. While the FOM of many of the presented integrated platforms is similar, the choice of material is yet critical. In particular, its transparency window and dispersion set the hard limitations to the SC achievable bandwidth as well as the targeted spectral range window for optimized operation. The material choice also dictates the hierarchy of the physical effects which drive the SCG process and affects all aspects and characteristics of the generated spectrum. All of these specific features will be highlighted in the performance summary tables included in the subsequent sections, which aim to review the SCG demonstrations that have been achieved in various integrated material platforms. It should be noted that the information in these tables is either directly taken from the corresponding publication or inferred/calculated from available values. Also, the definition of SC bandwidth, a key performance metric of SC, as will be briefly discussed in the concluding chapter, sometimes varies at −20 dB, −30 dB, or even −40 dB.

### 2.5 Fabrication of integrated nanophotonic waveguides

This section gives a brief overview of the fabrication process which is typically used to fabricate nanophotonic waveguide samples. It is kept very generic on purpose and should only provide enough details to be able to understand some advantages and limitations of integrated waveguides. While the exact process flow utilized to fabricate a nanophotonic waveguide varies from one publication to another, and while every material has different requirements and process steps and parameters, they mostly adhere to the process flow described here and all share the concept that the fabrication is done in cleanrooms using micro- and nano-fabrication techniques as they are known from the semiconductor industry. While these techniques can be applied on the wafer scale (typical sizes in research are 3 and 4 inches, or 75 and 100 mm, respectively), the process can also be done on the chip scale, if this seems to be advantageous.

**Figure 7:**
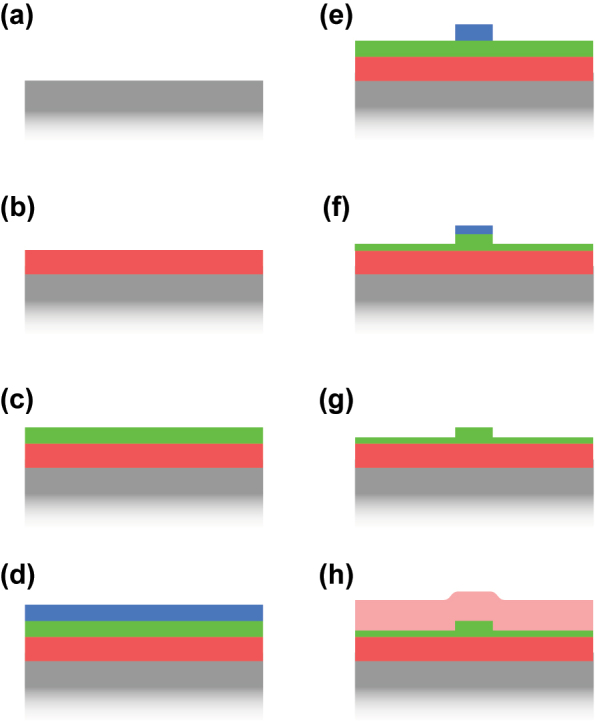
Generic fabrication process for nanophotonic waveguides. (a) The carrier wafer (grey), typically silicon. (b) Deposition of the bottom cladding (red) and (c) deposition of the waveguide layer (green). (d) And (e) lithography using a photoresist (blue). (f) Etching of the waveguide via the patterned photoresist mask. Here the waveguide is only partially etched. (g) Resist stripping and cleaning. (h) Top cladding deposition (light red).

The process flow starts with a carrier wafer, which is most commonly silicon (Figure 7(a), in grey). In order to provide a bottom cladding layer below the waveguide later on, a dielectric layer with low refractive index is required. For a silicon substrate this can be easily obtained by oxidizing the silicon of the wafer to silicon dioxide (red in Figure 7). If the carrier is already a low-index material such as sapphire or quartz, no additional layer is required. If it is neither a low-refractive index material nor silicon, silicon dioxide (or another cladding material) can be deposited via different techniques such as chemical vapor deposition (CVD) or physical vapor deposition (PVD). Onto this bottom cladding the optically nonlinear material (green) has to be deposited. As this layer will form the waveguide later on, the quality of the deposition is key to the performance of the device. Amorphous materials, such as silicon dioxide, silicon nitride, or silicon oxynitride, can be deposited with CVD processes. PVD is in principle also an option but is rarely used as it does not typically provide the required quality. If the material of the waveguide layer should be crystalline, different processes are employed. Most often these are variations of the crystal ion slicing (CIS, also known as Smart Cut) process, which is in particular used to transfer single crystalline layers of silicon and lithium niobate onto wafers with amorphous dielectric layer on top (e.g. silicon dioxide). In general, other processes to deposit high quality thin film could also be used, such as molecular beam epitaxy (MBE) or atomic layer deposition (ALD). Beside of a high quality film, the process used should also provide a good control over the thickness and uniformity over the wafer. The thickness of the film defines the height of the waveguides fabricated later and is therefore a critical parameter for the dispersion. Typical thicknesses are generally given by the vertical single mode criterion (∼*λ*/2*n*_eff_), which are in the range of 0.2–1 μm, even a few μm for certain MIR platforms.

Once the carrier wafer has the bottom cladding and the waveguide layer is deposited, the waveguide layer has to be structured. This is done via two steps, as for typical micro-fabrication processes. First, a lithography step structures a layer of photoresist (blue in Figure 7(d) and (e)). This photoresist acts as a protective layer where it remains on the wafer. Second, an etch step follows, which removes material from the waveguide layer where it is not protected by the photoresist. The initial photoresist is deposited onto the wafer via spin coating. The patterning of the photoresist is done by exposing the photoresist to either UV light or to an electron beam (ebeam) in dedicated machines. Exposure with an ebeam is typically very precise, it can achieve resolutions of around 10 nm. However, it is a serial process and the speed goes down with increasing resolution. UV lithography exists in several variations and can range from a simple contact lithography tool with a resolution of approximately 1 μm to high-end stepper tools with a resolution similar to that of an ebeam. The pattern, which describes where the photoresist should be exposed, is called a mask and is usually defined as a digital file containing the required 2D patterns. As the most commonly used file format is GDS or GDSII, the mask is sometimes also referred to as a GDS. The 2D geometries that are contained in the mask can be arbitrarily complex and in particular the waveguides can have bends, varying widths, and different lengths.

Once the photoresist is patterned, the wafer continues to the etching process (Figure 7(f)). The duration of the etching process determines how much of the unprotected waveguide material is removed. If the etching process lasts long enough, it will remove all the waveguide material where no photoresist is protecting it. This therefore defines a fully etched waveguide with a width and length that is described by the mask and a height which is defined by the thickness of the waveguide layer. In Figure 7 the waveguide is only partially etched. In most publications today dry etching steps are used relying on reactive ion etching (RIE) machines. Wet etching can be used and can lead to very good quality results but the required etchants are not available for all materials and wet etching results in sidewalls with an angle substantially smaller than 90°, whereas vertical sidewalls are mostly the preferred option to enable precise dispersion engineering. The key property of a good combination of lithography and etching step is a low sidewall roughness. Such low roughness results in lower optical losses due to scattering inside the waveguide.

After the etching step and some additional cleaning steps, the waveguide is fully defined (Figure 7(g)) and could already be used for SCG as the air on top of the waveguide does provide a good mode confinement inside the waveguide. However, the top of the waveguide is exposed to the environment. Therefore, often an additional top cladding is deposited, which is typically silicon dioxide but, similar as for the bottom cladding, it could be any other material with a lower refractive index than the waveguide material. Typical processes are again CVD processes.

As a last step, the waveguide ends have to be made accessible. In particular, if the waveguides are fabricated on a wafer, the wafer has to be broken down to smaller chips to work within the lab. Also here different processes exist. Manual cleaving of the wafer or breaking of the wafer is a simple but not very precise method. More common is the dicing of the wafer using blade dicing or laser dicing. Other processes rely on additional etching of the layers to define the facets of the photonic chips.

## 3 Supercontinuum generation in Si-photonics

Silicon photonics has attracted a large research interest, mainly motivated by the fact that it is based on the same fabrication process flow as complementary-metal-oxide-semiconductor (CMOS) electronics. In this section we review the SCG work based on the three main Si photonics platforms: Si, SiN, and SiGe.

### 3.1 Silicon based devices

**Figure 8:**
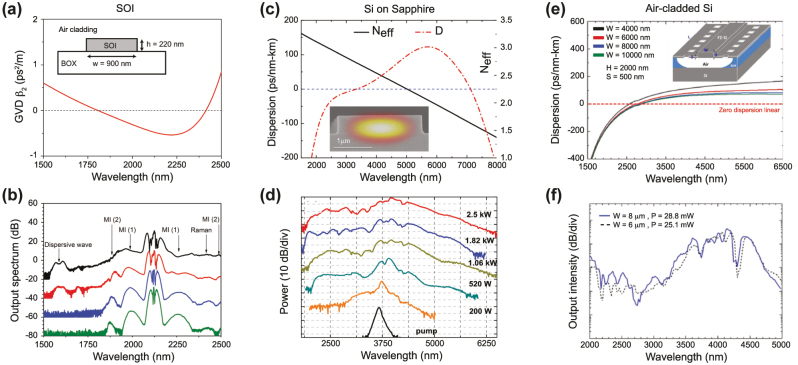
SCG in Si platforms. (a) Calculated GVD as a function of wavelength, inset: schematic of the waveguide and (b) measured output spectrum for coupled input peak power: 3.1 W (green), 4.3 W (blue), 7.9 W (red), and 12.7 W (black). The spectra are vertically offset by multiples of 20 dB for clarity. (c) Calculated dispersion and effective index for SOS nanowire with a cross section of 2.4 μm by 0.48 μm shown in inset and (b) measured output spectrum with input peak power ranging from 200 W to 2.5 kW. (e) Calculated dispersion for suspended SI waveguide of thickness (H) 2 μm, a slab thickness (S) of 0.5 μm and various height (H), inset: schematic of the suspended silicon waveguide, and (f) measured output spectrum for two widths. Panels (a) and (b) adapted from [64]. Panels (c) and (d) adapted from [67]. Panels (e) and (f) adapted from [68].

Silicon, with its very high nonlinear index *n*_2_ and large refractive index, allowing for tight confinement of light in nanometer scale structures, appears as an excellent material choice for SCG. The waveguide geometry used in most work is that of silicon on insulator (SOI) where the Si waveguide is patterned on a buried oxide layer of SiO_2_, as seen in Figure 8(a). Such common structure, with the waveguide either fully clad in SiO_2_ or left uncladded, leverages the high index contrast (∼2) between Si and SiO_2_ for creating tightly light confining waveguides (*A*_eff_ of roughly 0.1 μm^2^) with relatively low propagation losses in the few dB/cm. Hsieh et al. [63] reported the first pulse spectral broadening in a silicon waveguide in 2007. The 0.52 μm × 0.22 μm waveguide had an expected high *γ* (6 × 10^4^ W^−1^ m^−1^) and a predicted ZDW near 1300 nm, where the pump was positioned. However, the measured broadening was limited to 3/10 of an octave, severely limited by two-photon absorption (TPA) of Si in the telecom band. As to eliminate this crippling effect, the large majority of subsequent work has relied on pumps in the short-wave infrared (near 2 μm, which falls in the thulium emission band) or deeper in the MIR using OPOs. Kuyken et al. [64] showed the first demonstration of an SC going beyond the telecom band. Relative long pump pulses (2 ps) were used such that MI was the main broadening mechanism and the overall spectral span remained limited (Figure 8(b)). The first octave spanning SC was demonstrated a few years later by Lau et al. [65]. The waveguide was pumped very close to the maximum GVD using a 2.5 μm pump in the fs regime. The broadening, there dominated by soliton fission, showed clear DW on both the blue and red detuned side of the pump. The phase coherence nature of a part of such SCG, measured in the short-wave infrared region, was confirmed in [66]. Some demonstrations also proved that it is possible to generate SC in an SOI platform operating up to the short wavelength edge of the SOI transmission window, near 1 μm, while relying on fiber laser pumping. A first attempt in a hydrogenated amorphous silicon waveguide leveraged a commercially available Thulium doped mode-locked fiber laser. The generated MI driven SC was limited in bandwidth owing to the relatively long 1.24 ps pulses together with the low available peak power. In [57], a 100 fs 1550 nm fiber laser Raman shifted in a highly nonlinear fiber to 1.95 μm was used to pump a short SOI waveguide whose dispersion was engineered as to have the pump positioned close to the first ZDW located in the near-IR. This work proved that an octave spanning near-IR and fully coherent SC can be efficiently generated in SOI.

Despite these few near-IR works, the obstacle posed by TPA has pushed octave-spanning SCG research in Si into longer wavelength region (*λ* ≥ 1950 nm). At this point, the silica substrate starts being the limiting factor, owing to its fast increasing absorption losses beyond 2.5 μm. To circumvent silica losses, two main approaches are pursued: replacing the silica substrate with sapphire or suspending the Si waveguide. The first Si on sapphire (SOS) based waveguides were reported in 2010 with propagation losses of 4.3 dB/cm at 4.5 μm [69]. The nonlinear optical performance of SOS was established by Singh et al. in 2015 [67]. The waveguides were fabricated by epitaxially growing 0.5 μm of silicon on a sapphire substrate. To smooth the surface of the waveguide as to minimize scattering losses, the devices were treated with repeated chemical oxidation and oxide stripping. The waveguide was dispersion engineered as to achieve low and anomalous dispersion near the 3.7 μm pump, which required a large width (Figure 8(c)). With a coupled peak power of 2.5 W, the continuous SC spanned 2–5.58 μm as seen in Figure 8(d).

Similar broadening was experimentally obtained in a suspended all-air-cladding structure a few years later. A wet etching method was used to fabricate the suspended waveguides shown in Figure 8(e) [68]. Holes of 1 μm × 2 μm were made by e-beam lithography and reactive ion etching (RIE) around the waveguide structure for wet etching. The oxide layer under the waveguide was removed by dipping the substrate in a 5:1 buffered hydrofluoric acid. The 0.5 μm slab was thick enough to avoid deflection of the Si membrane and to guarantee mechanical stability as to generate an SC spanning 2–5 μm from a 4 μm pump. This wet etching technique allows for the necessary flexibility in dimensions to tailor the dispersion. The performance of such suspended structure was further improved by significantly lowering the coupling losses utilizing fork-shaped couplers and lowering the propagation losses to 2 dB/cm at the pump wavelength [58]. The 5 mm suspended waveguides are fabricated based on 0.7 μm thick fusion bonded Si membranes provided by an SOI wafer [70]. The air trenches underneath the waveguides are etched in a blank Si wafer prior to bonding and are designed to avoid leakage loss of the generated light. The long wavelength ZDW can be tailored from 3.5 to 5 μm by varying the waveguide width. With a waveguide width of 3.25 μm and a height of 0.7 μm, a continuous SC spanning 2–7.7 μm, the long wavelength onset of Si absorption, was demonstrated, with a low enough soliton number to guarantee coherence.

**Figure 9:**
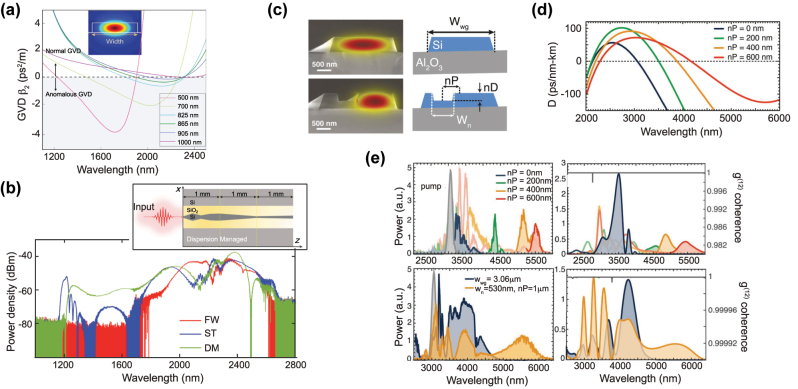
Advanced designs in Si platforms for SCG. (a) Computed GVD of the fundamental quasi-TE mode of 0.22 μm thick SOI waveguides with various fixed widths, inset: fundamental transverse mode in the 0.825 μm wide waveguide at 2200 nm. (b) Measured output spectra recorded for the dispersion managed (DM), single taper (TP), and 0.825 μm fixed width (FW) waveguides and schematic geometry of the DM structure. (c) SEM images and cross-section diagram of strip and notch SOS waveguides. (d) Calculated GVD profiles of the notch SOS designs. (e) Measured output spectra of notch waveguides (top), and octave span broadening in a strip (blue) and notch (orange) waveguides (bottom). Experimental results are shown on the left and simulations on the right together with calculated coherence. Panels (a) and (b) adapted from [72]. Panels (c)–(e) adapted from [73].

More advanced designs have also been suggested, with the primary goal to better control and shape the generated SC. A cascaded design based on the concatenation of waveguides with different cross-sections as to vary the dispersion along the length of the devices was proposed and demonstrated in [71]. The authors showed that such design can be used for selective spectral enhancement. They also leveraged the strong birefringence of the design by pumping simultaneously with both quasi transverse electric (TE) and transverse magnetic (TM) modes. Following a similar trend, the SCG performance of fixed width, single taper, or dispersion managed (i.e. varying width, here over 7 different sections) waveguides was compared in [72]. It was shown that more complex designs such as dispersion managed, can provide a significant advantage in terms of broadening, reach and flatness as seen in Figure 9(a) and (b). They explain this by the trapping of the blue emitted DW, which is initially efficiently generated close to the pump, and then a continuous blue shift as the DW and the main compressed pulse propagate together in a tapered section of the dispersion managed waveguide. This comes from the optimization of the dispersion profile, which allows this DW to be continuously group-velocity matched with the pump. Note that while similar approaches for dispersion management along the propagation direction were first and advantageously developed in fiber-based SCG by concatenating fiber sections with varying dispersion profiles, their implementation in chip-based waveguides provides even more flexibility in the design and a refined control of the dispersion properties during the lithography step. A notched design for the SOS platform was proposed in [73] as to provide an extra degree of freedom for dispersion engineering, which was used to experimentally show a controlled SC (Figure 9(c)–(e)). Very recently, the use of suspended silicon metamaterial waveguides was proposed as a way to efficiently control the position of the DW [74]. The waveguide is composed of a central strip and a lateral metamaterial-grating cladding. This cladding is not only used to selectively remove the silica under-cladding, but also for dispersion engineering. The optimization of the metamaterial waveguide geometry was demonstrated in the context of independent control of the position of the short and long-wavelength DWs. To conclude this brief review of SCG in Si based waveguides, the detailed geometry, linear and nonlinear properties of the waveguides exploited in the various works described above, as well as the resulting SC performance obtained, are summarized in Table 3, also highlighting, for comparison, both the corresponding peak and averaged coupled pump powers $\left(P_{\text{P}}^{\text{A}}\right)$ , needed to generate the related SC.

**Table 3:** Summary of the main SCG results presented and obtained in Si waveguide based devices. The last four rows relate to designs departing from standard rectangular cross-section geometries.

Platform	Width × height, length	Pump (nm)	*γ* (mW)^−1^	*α* (dB/cm)	Coupling loss	$P_{\text{P}}^{\text{C}}$ (W) ( $P_{\text{A}}^{\text{C}}$ (mW))	Bandwidth	Year
SOI [64]	0.9 μm × 0.22 μm, 20 mm	2120	150	2.5	10 dB/facet	12.7 (1.9)	1.525–2.524 μm	2011
SOI [65]	1.21 μm × 0.32 μm, 20 mm	2500	58.5	N.A.	9 dB/facet	15 (0.4)	1.51–3.67 μm	2014
SOI [57]	0.92 μm × 0.315 μm, 5 mm	1950	142	1.5	N.A.	360 (3.6)	1.06–2.4 μm	2018
SOS [67]	2.4 μm × 0.48 μm, 16 mm	3700	8.86	1	9 dB/facet	2500 (16)	2–5.58 μm	2015
Suspended Si [68]	6 μm × 2 μm, 12 mm	4000	N.A.	5	8 dB/facet	3300 (12)	2–5 μm	2018
Suspended Si [58]	3.25 μm × 0.7 μm, 5 mm	3060	N.A.	2	1.5 dB/facet	1100 (9.3)	2–7.7 μm	2019
SOI [71]	0.92 μm × 0.315 μm + 1.07 μm × 0.315 μm 5 + 5 mm	1950	N.A.	1–1.5	N.A.	100 (2)	1.3–2.3 μm	2019
SOI [72]	[0.92–0.9] μm × 0.22 μm, 3 mm	2260	N.A.	N.A.	24.5/8.3 dB (in/out)	47 (0.3)	1.2–2.3 μm	2020
SOS [73]	3.45 μm × 0.66 μm, 10 mm	3060	N.A.	5–7	6.7 dB/facet	1200 (12)	2.5–6.2 μm	2018
Suspended Si [74]	3.6 μm × 0.7 μm, N.A.	3500	N.A.	1–2	12 dB/facet	1000 (0.2)	1.53–7.8 μm	2022

### 3.2 Silicon nitride based devices

As mentioned in the previous section, TPA and relatively high linear losses severely limit the use of Si in the near-IR range. In recent years, SiN, particularly in its stoichiometric form, has appeared as a go-to platform for linear and nonlinear integrated optics. It is well known as a dielectric insulator and was initially used as a platform for integrated linear optics [75]. High-confinement Si_3_N_4_ waveguides have then been suggested as a nonlinear alternative to Si, since Si_3_N_4_ is characterized by a large bandgap close to 5 eV and a wide transparency window from 0.350 to 7 μm. However, Si_3_N_4_ has a nonlinear refractive index an order of magnitude smaller than Si, as well as a lower refractive index. While this results in reduced values of *γ* with respect to that of SOI waveguides, the linear losses are also significantly lower, and higher peak and average powers can be coupled to Si_3_N_4_ waveguides, as seen in Table 4. But most importantly, the particularly crippling effect of TPA and the limiting absorption edge of Si at 1.1 μm do not apply.

**Table 4:** Summary of the main SCG results presented and obtained in silicon nitride based waveguides. The last three rows relate to waveguides with different material composition from the stoichiometric Si_3_N_4_.

Platform	Width × height, length	Pump (nm)	*γ* (mW)^−1^	*α* (dB/cm)	Coupling loss	$P_{\text{P}}^{\text{C}}$ (W) ( $P_{\text{A}}^{\text{C}}$ (mW))	Bandwidth	Year
Si_3_N_4_ [76]	1.1 μm × 0.72 μm, 43 mm	1335	1.2	0.2	8.3 dB/facet	900 (14.4)	0.625–2.025 μm	2012
Si_3_N_4_ [77]	0.5 μm × 0.3 μm, 10 mm	795	7	11 (at 0.6 μm)	8.5 dB/facet	974 (7)	0.488–0.978 μm	2015
Si_3_N_4_ [78]	0.9 μm × 0.69 μm, 7.5 mm	1060	3.25	0.7	N.A.	391 (37)	0.6–1.7 μm	2015
Si_3_N_4_ [79]	1 μm × 0.9 μm, 6 mm	1560	1	0.5	5.4 dB/facet	11000 (52)	0.526–2.584 μm	2017
Si_3_N_4_ [80]	2 μm × 0.75 μm, 55 mm	2100	0.74	0.5	6 dB/facet (TE), 11 dB/facet (TM)	12000 (25) (TE), 4500 (9) (TM)	0.54–2.670 μm (TE), 1.65–2.3 μm (TM)	2021
Si_3_N_4_ [81]	2.7 μm × 0.69 μm, 200 mm	1560	0.7	0.029	8 dB/facet	6560 (82)	1.010–2.020 μm	2022
Si_3_N_4_ [56]	1.1 μm × 2.3 μm, 5 mm	1550	N.A.	0.2	3 dB/facet	2200 (20)	0.55–4.0 μm	2018
Si_3_N_4_ [82]	1.175 μm × 2.29 μm, 5 mm	2090	0.37	0.2	5.5 dB/facet	6800 (13.6)	0.5–4.0 μm	2019
Si-rich SiN [83]	1.65 μm × 0.7 nm, 10 mm	1555	5.7	1.35	6.5 dB/facet	1000 (9)	0.82–2.25 μm	2016
Deuterated SiN [84]	2.7 μm × 0.86 μm, 3.9 mm	1560	N.A.	0.31	1 dB/facet	17000 (140)	0.7–2.2 μm	2018
N-rich SiN [85]	0.7 μm × 0.6 μm, 3 mm	1200	1	0.4	7 dB/facet	3800 (0.5)	0.40–1.6 μm	2020

Most of the work in Si_3_N_4_ relies on the use of standard rectangular cross-section waveguide geometries, where the core width and height are varied to shape the dispersion. However, light confinement and dispersion engineering historically proved difficult with Si_3_N_4_ due to the tensile film stress which, for a long time, limited the achievable thickness to less than 0.25 μm, i.e. not suited for nonlinear operation beyond the telecom band nor to easily reach anomalous dispersion regime underpinning straightforward SCG exploiting soliton dynamics. Recognizing the high potential of the platform, significant efforts were put in maturing fabrication processes as to allow the use of thick films. Nowadays low loss layers thicker than 500 nm have been achieved by both plasma-enhanced chemical vapor deposition (PECVD) [21, 89, 90] and low-pressure chemical vapor deposition (LPCVD) [91–93], removing the final barriers towards nonlinear optics in silicon nitride. While high temperature annealing is necessary with LPCVD deposition, extremely low propagation losses have been reached and the large majority of nonlinear demonstrations rely on such fabrication method. Slightly higher losses are exhibited from PECVD deposition, but the low temperature processing is advantageously back-end of line (BEOL) process compatibility.

**Figure 10:**
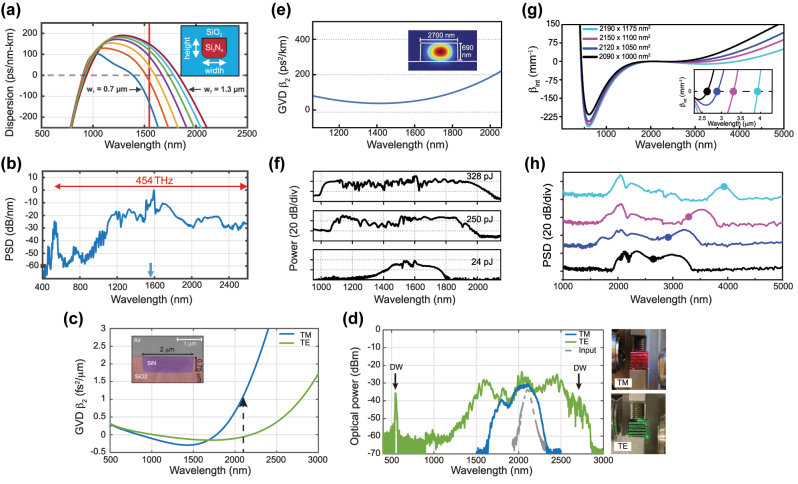
SCG in SiN platforms. (a) Simulated dispersion for the fundamental TM-mode in waveguides with a height of 900 nm and different widths, inset: cross section, and (b) output spectrum from 1000 nm × 900 nm with pump wavelength indicated by the arrow. (c) Simulated GVD for the fundamental TE and TM mode, dotted arrow indicates pump wavelength, inset: SEM image of the waveguide and (d) output spectra for TE (anomalous) and TM (Andi) mode coupling and attenuated input pump pulse, photographs of the waveguide taken from the top showing red scattered light for TM excitation (third harmonic of the pump initiated from intermodal phase matching right from the input of the waveguide), and green scattered light for TE excitation (DW generated a few mm after the beginning of the propagation, following soliton fission). (e) Simulated GVD of the waveguide with a geometry of 2700 nm × 690 nm showing all normal dispersion, inset: Simulated TE mode profile, and (f) measured output spectrum using different energies for the input pulses. (g) Integrated dispersion for four waveguide geometries, inset: expected MIR phase matching points, and (h) experimental output spectra (same color convention as for (g)). Panels (a) and (b) adapted from [79]. Panels (c) and (d) adapted from [80]. Panels (e) and (f) adapted from [81]. Panels (g) and (h) adapted from [82].

In 2012, Halir et al. demonstrated what was at the time the broadest SC in a CMOS fabrication compatible chip [76]. Using a near-IR pump at 1335 nm from an OPO, they generated a 1.6 octave spectrum in a 43 mm long Si_3_N_4_ waveguide. The pump was positioned in between the two ZDWs of the waveguides such that the SC was bound by two DWs, between 0.625 and 2.2025 μm. By optimizing the dispersion and relying on thicker Si_3_N_4_ layer of 0.9 μm while maintaining low propagation losses, the long wavelength ZDW could be pushed deeper towards the MIR as the waveguide width was increased up to 1.3 μm [79] (Figure 10(a)). A 1560 nm pump was then well positioned close to the maximum value of GVD such that a 2 octave spanning SC was generated. The total extent of the spectrum could however not be fully determined due to the limited operating range of the optical spectrum analyzer used (Figure 10(b)). Unlike the work on Si reviewed in the previous section which was restricted to exploiting anomalous dispersion waveguides, Si_3_N_4_ also shows excellent performance in terms of ANDi SCG, a less studied approach in waveguides since a low and flat all-normal dispersion at telecom wavelengths is typically more challenging to achieve in integrated designs. In [80], ANDi SCG was demonstrated by engineering the dispersion of the polarization modes in an air-clad Si_3_N_4_ waveguide (Figure 10(c)). The ANDi SC obtained from pumping the TM mode showed the expected smooth spectrum without the fine spectral structure or large dips that are seen when pumping the TE mode in the anomalous dispersion regime, a typical signature of SCG driven by soliton dynamics, as shown in Figure 10(d). However, the broadening for the ANDi SC was limited owing to the large value of GVD at the pump wavelength, such that the fs pump pulse quickly broadened along the propagation direction. In [81], the authors fabricated an optimized ANDi Si_3_N_4_ waveguide, with a low and flat GVD value near 1550 nm (Figure 10(e)), based on a subtractive processing method [94]. Similar to [80], the cross-section of the waveguide is relatively large resulting in a *γ* less than 1 m^−1^ W^−1^. However, as the propagation loss with such fabrication method is extremely small (less than 3 dB/m), long waveguides can be leveraged to compensate for this low nonlinearity. This however imposes even more constraints on the value of GVD. By balancing all these aspects, the achieved ultra-low loss, 20 cm long three-spiral waveguide enabled coherent broadening of ps pulses at high repetition rate. When using pulses with higher energy and femtosecond duration, they could observe optical wave breaking on chip, resulting in an octave spanning ANDi SC, as seen in Figure 10(f).

Another attribute of silicon nitride is its transparency from the visible to the MIR (near 5 μm). The SC extension to the MIR is however made difficult by the large cross-section required for MIR light propagation, but also dispersion engineering which plays a key role in extending the long-wavelength portion of the SC. In order to allow efficient light conversion over a large frequency-span, soliton-induced DW generation is the most appropriate mechanism. As previously mentioned, in this context, the emergence of a ZDW at the longer wavelength side of the pump source is required, which is also challenged by the strong anomalous material dispersion. This implies an operation approaching the mode cut-off region of air-clad waveguides, or alternatively, in the absence of cut-off for fully clad waveguides, some loss of confinement towards the MIR and, consequently, significant absorption in the cladding. The possibility to grow high quality Si_3_N_4_ films thicker than 1 μm and improvement in the fabrication process of large cross-section waveguides, led to the first direct generation of light beyond 3 μm, from an erbium-doped fiber laser through DW generation [56]. Using a waveguide with a width of 1.11 μm and a height of 2.3 μm together with a pump at 1550 nm, an SC spanning up to 4 μm was measured. The MIR light showed high phase coherence and a frequency comb nature. However, the outstanding problem in this first demonstration was the low conversion efficiency and power beyond 3 μm. Indeed, reaching efficient DW generation beyond 3 μm is still difficult in CMOS platforms directly pumped by fiber lasers that have limited power. The larger the spectral coverage, the lower the power transferred in the targeted region. Additionally, the SCG process can convert a non-negligible portion of the pump energy over unwanted spectral bands. In [82], by optimizing the waveguide geometry (Figure 10(g)), and by leveraging the physics of DW generation, the conversion efficiency within the 3–4 μm wavelength improved significantly, reaching 35% at 3.05 μm and 20% at 3.95 μm (Figure 10(h)). For this work, the pump wavelength was shifted to the short-wave infrared near 2 μm, as to favor dispersion for targeted long wavelength operation, while still relying on a commercial fiber laser.

**Figure 11:**
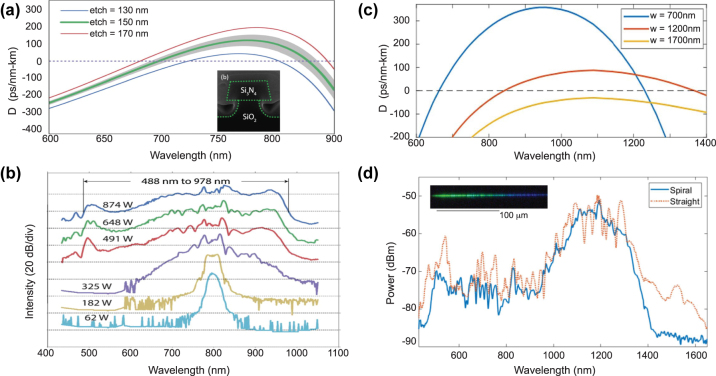
Short wavelength SCG in silicon nitride platforms. (a) Simulated dispersion of the under etched waveguide for various values of the waveguide under etching, inset: SEM picture of the waveguide cross-section, and (b) output spectra for various input coupled peak power coupled into the waveguide. (c) Calculated dispersion in an N-rich SiN_*x*_ waveguide for different widths, and (b) experimental spectra for the 8.6 mm long, 1.2 μm wide spiral waveguide, and 3 mm straight waveguide, inset: optical images of the straight waveguide. Panels (a) and (b) adapted from [77]. Panels (c) and (d) adapted from [85].

The potential of the Si_3_N_4_ platform for operating in the visible was confirmed in [77], with the demonstration of an octave spanning SC covering 0.488–0.978 μm when pumped at 795 nm as seen in Figure 11(a) and (b). In order to reach the visible range, one of the main hurdles in Si_3_N_4_ is to achieve the required dispersion for SCG: the strong normal material dispersion at short wavelengths due to the proximity of the bandgap has to be compensated in order to reach anomalous dispersion regime sustaining efficient and broad SC generation. A Si_3_N_4_ waveguide of small cross section was fabricated, and the dispersion was significantly trimmed following a previously proposed method of partially under-etching the silicon oxide underneath the waveguide core [95]. Waveguide loss over the spectral region of interest was estimated around 11 dB/cm, compensated by the improved *γ* obtained through the reduced effective area as well as the short operation wavelength. In [96], it was shown that a waveguide with a height of 1 μm and width of 0.8 μm resulted in shifting the first ZDW, enabling efficient SCG with a 1064 nm fiber laser pump. The SC extended from 470 to 2130 nm, with a significant improvement on the blue side of the spectrum.

A last direction of research aimed at optimizing the performance of silicon nitride for SCG, is to modify the material composition. All the previously mentioned results were obtained from Si_3_N_4_. An interesting feature of Si_*x*_N_*y*_ films is that their composition can be adjusted during the deposition process, such as to have a richer Si (for Si-rich SiN) or richer N (for N-rich SiN) content. Some of the initial investigation at increasing the Si content was motivated by the reduction of the stress as to allow for thicker waveguides [97]. For nonlinear optics, an increase in the Si content also results in an increased nonlinear index as well as the possibility to bandgap engineer the material [98–101]. With 65% Si content, a refractive index of 2.1, bandgap of 2.3 eV, and a nonlinear refractive index *n*_2_ = 1.4 × 10^−14^ cm^2^/W, about 5 times larger than Si_3_N_4_, were measured while maintaining low propagation loss at around 1 dB/cm. The first demonstration of SCG in Si-rich SiN used back-end CMOS compatible PECVD deposition with a Si content almost three times higher than stoichiometric silicon nitride [102]. As such, the nonlinear parameter of the waveguide was extremely high (around 550 W^−1^ m^−1^) but the losses were also significant (10 dB/cm). Using a 1550 nm pump in a 7 mm long waveguide, the obtained SCG spanned from 1.3 to 1.7 μm, limited by the dispersion of the waveguide and the loss on the short wavelength side. With a slightly richer Si content, the performance was improved in [103], and although the nonlinearity was not as high as in [102], the lower losses and improved dispersion, with, at the time, a much simpler fabrication procedure owing to the lower tensile stress, allowed to obtain an octave spanning SC. Finally, modifying the composition can also be employed towards the end of avoiding the need for high temperature annealing typically used for Si_3_N_4_ films. The work in [104] put forward the true CMOS compatibility of their platform as the silicon nitride films were deposited using inductively coupled plasma chemical vapor deposition at a temperature of 250 °C, which is compatible with back-end CMOS processes that are limited to temperatures below 400 °C. PECVD is indeed a straightforward processing technique with high repeatability and low temperature requirements. However, the losses of the deposited Si_3_N_4_ films are high due to the N–H bonds, since the hydrogen content is significant in un-annealed thin films. Reducing the N–H bonds content has been pursued to reduce the losses. In [84], the authors use isotopically substituted precursors during deposition to modify the bond energy of the N–H overtone. More specifically, they employ deuterated silane (SiD) instead of conventional silane (SiH) which shifts the absorption band from 1.5 μm to 2 μm. In such deuterated SiN waveguides pumped at telecom wavelength, the loss was measured to be as low as 0.31 dB/cm, enabling the demonstration of an octave spanning SC. As the refractive index of silicon nitride decreases with an increase of nitrogen [105], the N-rich SiN appears well suited for operation in the O-band (1260–1360 nm), also motivated by the fact that N–H bonds do not affect the propagation loss in the O-band such that low-temperature PECVD deposition is not detrimental in this case. This was validated in [85], where low-linear loss N-rich SiN films deposited through a simple back-end of line-compatible process enabled the demonstration of two-octave spanning SC covering the visible and the O-band (Figure 11(c) and (d)).

It is evident that a large number of publications on SCG in silicon nitride have been published in the last years, pushed by the maturing of the fabrication techniques and of the intrinsic material qualities, resulting in some of the lowest propagation losses achievable in any integrated nonlinear platform. The most recent silicon nitride SCG developments have aimed, on the one hand, at extending the wavelength coverage beyond the standard telecom wavelengths, either pushing towards the MIR or the visible by leveraging material composition and advanced dispersion engineering, and on the other hand, at improving the compatibility with BEOL CMOS process flows.

### 3.3 Germanium and silicon germanium based devices

The high nonlinear index [23], and the CMOS compatibility of SiGe and Ge make Ge-based platforms ideal candidates for integrated nonlinear photonics. In particular, because of the wide transparency window of Ge in the MIR (from 3 to 15 μm) [24], Ge-based platforms are well suited for MIR SCG, but not directly competing with the group IV material platforms presented in the two previous sections (Si and silicon nitride) that tend to operate at shorter wavelengths. So far, in terms of SCG, most of the results have been obtained in waveguides with a SiGe core, whereas pure Ge is lagging behind, mainly because of fabrication challenges related to the large lattice mismatch between Ge and Si.

After the first studies on the nonlinear properties of SiGe waveguides [23, 106–108], their potential for SCG was confirmed by the first experimental demonstration in 2015. Ettabib et al. demonstrated SCG in the short wavelength infrared, from 1.45 to 2.79 μm, in a dispersion-engineered graded-index (from 0 to 42% Ge concentration) SiGe/Si waveguide, by pumping in the anomalous dispersion with 90 fs pulses at 2.4 μm [86]. The device was grown by reduced-pressure chemical vapor deposition on an SOI wafer and patterned by photolithography and etching. The authors estimated a conversion efficiency of 16%. The dominant loss mechanisms were attributed to linear losses (2 dB/cm) and nonlinear losses given by three-photon absorption (3 PA) and free carrier effects. The same year, Carletti et al. observed a drastic decrease of 3 PA beyond 3.8 μm for Si_0.6_Ge_0.4_/Si waveguides [106, 107]. These studies paved the way to the demonstration, in step-index SiGe/Si waveguides pumped at ∼4 μm (i.e. beyond the 3 PA threshold), of the first octave spanning SCG, up to 8.3 μm, by Sinobad et al. [22]. The waveguides were fabricated on a 200 mm CMOS pilot line, using regular processes. First, the SiGe layers were grown on top of a Si(001) substrate by reduced pressure-chemical vapor deposition. The waveguides were then patterned using deep ultraviolet photolithography followed by a deep reactive ion etching process. The air-clad waveguide geometry enabled to achieve single-mode operation and, more critically, anomalous dispersion at the pump wavelength. The use of a longer pump wavelength, along with the lower propagation losses (below 0.6 dB/cm from 3.5 to 5 μm, and as low as 0.23 dB/cm at 4.15 μm), allowed obtaining two major results. First, the experimental demonstration of a 1.4 octave spanning SC, extending from 3 to 8.3 μm in a large cross-section (6 μm wide and 4.2 μm high) waveguide with up to 12.5 mW converted on-chip power, corresponding to 50% conversion efficiency. The long wavelength limit was attributed to the absorption from the silicon substrate, which starts to be non-negligible beyond 8.3 μm. Second, a slightly narrower but efficiently generated SC, extending from 2.63 to 6.18 μm, was demonstrated in a smaller cross-section waveguide (3.75 μm wide and 2.7 μm high). This smaller cross-section provides a higher gamma parameter of 0.63 m^−1^ W^−1^ compared to 0.3 m^−1^ W^−1^ for the large cross-section waveguide, thereby boosting the conversion efficiency. The waveguide was designed to exhibit a narrow anomalous dispersion region enclosed between two normal dispersion regions. This enabled the generation of an SC that retained high coherence at the extreme parts of the spectrum, at frequencies distanced by one octave, with potential applications for f-to-2f frequency stabilization [109]. In addition, the smaller cross-section made it possible to control the properties of the generated SC via post-processing dispersion trimming by heterogeneous integration of coating materials [87].

**Figure 12:**
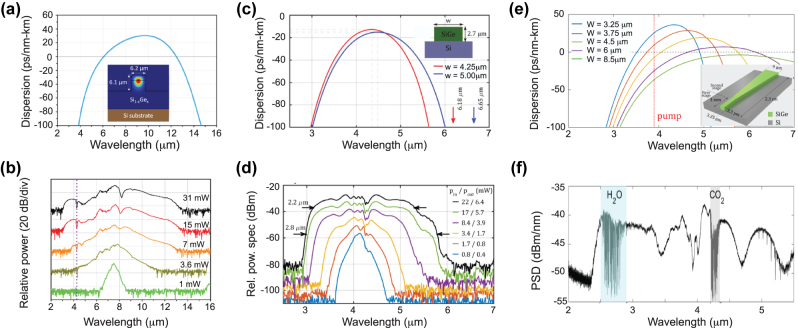
SCG in Ge based platforms. (a) Simulated dispersion in a graded index SiGe/Si waveguide, inset: waveguide schematic with mode profile at 7.5 μm. (b) SCG for different pump powers. (c) Simulated dispersion in a step index SiGe/Si waveguide, inset: SEM image. (d) SCG for different pump powers showing ANDi regime. (e) Simulated dispersion in a two-stage SiGe/Si waveguide, inset: waveguide schematic. (f) SCG in the two-stage SiGe/Si waveguide. The absorption dips from water vapor and CO_2_ are highlighted by the shaded areas. Panels (a) and (b) adapted from [59]. Panels (c) and (d) adapted from [15]. Panels (e) and (f) adapted from [88].

The wide transparency window of SiGe compounds was eventually fully leveraged in 2020 by the group of Marris-Morini, with the demonstration, in a graded index SiGe/Si platform, of SCG from 3 to 13 μm (Figure 12(a) and (b)) [59]. The SiGe layer, with Ge fraction linearly increasing from 0 to 0.79, was grown by low-energy PECVD, and then patterned by laser lithography and inductively coupled plasma-reactive ion etching for waveguide fabrication. The graded index configuration mitigated the absorption from the silicon substrate, resulting in extending the SC to longer wavelengths. However, the on-chip SC power was not reported. We note that this record bandwidth, showing the full potential of the SiGe platform for SCG, was obtained at the cost of increasing the pump wavelength to 7.5 μm, which may hinder the development of a fully integrated device, since pulsed lasers at shorter wavelengths are easier to miniaturize.

These demonstrations proved that SiGe/Si is well-suited for the generation of broadband SCs in the MIR, outperforming in this range other Si-based platforms, although requiring a long wavelength pump. For many applications, however, in addition to a wide spectral bandwidth, a high degree of coherence and single pulse spectrum are required. As previously described, these features can be obtained in waveguides exhibiting an ANDi dispersion profile, at the price of a reduced bandwidth. By adapting the waveguide’s cross-section, Sinobad et al. achieved in 2020 ANDi operation in the step index Si_0.6_Ge_0.4_/Si platform (Figure 12(c)) [15]. The authors reported a fully coherent SC extending from 2.8 to 5.7 μm for 39 mW coupled average power (Figure 12(d)). The inferred on-chip SC power (∼15 mW) was comparable to the one previously reported in the waveguides operating in the anomalous dispersion regime [22]. In both dispersion regimes, the spectral bandwidth saturated after a propagation distance of ∼20 mm, as a consequence of the waveguide dispersion, and of the linear and nonlinear losses. Recently, the same group proposed a two-stage design (Figure 12(e)), in order to combine the broad bandwidth provided by the anomalous dispersion with the flatter spectral shape typical of SCG in the ANDi regime [88]. The authors demonstrated a 40% increase in the SCG efficiency with such a dispersion-managed waveguide, and harnessed the improved spectral properties of the SC for the simultaneous detection of water vapor and CO_2_ (Figure 12(f)).

Although the strong potential of the SiGe/Si platform has been demonstrated for on-chip MIR SCG, this platform exhibits a low index contrast between the SiGe core and the Si substrate (∼0.12 at 4 μm). This represents a shortcoming, since large waveguide cross-sections are accordingly required, in turn lowering both *γ* and the SCG conversion efficiency. To some extent, the characteristics of these SiGe/Si waveguides make them more similar to optical fibers, yet more robust and easier to dispersion engineer. In addition, to go beyond 8.5 μm, graded index SiGe is required, which leads to even larger modal area and lower efficiency. In this context, pure germanium-on-silicon (Ge/Si) waveguides have appeared as another promising candidate to cover the longer 8–12 μm band. Firstly, Ge outperforms every other group IV material in terms of transparency window deeper in the MIR, since it transmits light to beyond 12 μm. Secondly, the higher refractive index difference between the Ge core and the Si substrate (∼0.7 between 3 μm and 10 μm) allows to design waveguides with a smaller cross-section and tighter mode confinement in the Ge core material. Combined with the ten times higher Ge third-order nonlinearity than in Si, as is theoretically expected [23], this should be beneficial for improving the SCG performance. Until recently, however, no SCG had been reported in pure Ge waveguides. This is mainly due to the difficulty of growing high-quality thick Ge layers (because of the large Ge/Si lattice mismatch), as required to obtain a waveguide dispersion suitable for SCG in the MIR. Recently developed Ge/Si waveguides have been shown to transmit light with relatively low loss (a few dB/cm) up to 11–12 μm [110, 111]. This less mature platform does not reach the performance of the ultralow loss SiGe/Si waveguides discussed above yet, while its nonlinear properties are still not well known. After the first theoretical studies showing that Ge would be suitable for nonlinear devices (*n*_2_ = 2.55 × 10^−17^ m^2^/W, and no TPA beyond 3.2 μm [23]), the nonlinear properties of Ge have been experimentally investigated by means of z-scan measurements [26, 27]. There is however significant discrepancies between the latter two studies. In particular, ref. [27] reported a hundred times higher value of 3 PA in the 3.7–5.2 μm wavelength range as compared to ref. [26]. The single experimental demonstration of SCG in a pure Ge waveguide reported to date showed that the amount of 3 PA at 4.6 μm was indeed particularly detrimental, thus preventing to fully exploit the whole transparency window of Ge so far [25]. In this work, the authors measured an SC extending from 3.53 to 5.83 μm in TE polarization, and from 3.33 to 5.55 μm in TM polarization, with milliwatt level on-chip average power in a 2.57 μm × 4.46 μm Ge/Si ridge waveguide operating in the anomalous dispersion regime pumped at 4.6 μm. As expected, the retrieved *γ* parameter was around three times higher than that of SiGe/Si waveguides. The long wavelength limit of the SC was attributed to the absorption from free-carriers generated by 3 PA. Indeed, in the particular case of Ge, free carrier absorption sharply increases beyond 6 μm. To fully exploit the transparency window of Ge, it is thus essential to limit the free carrier density by keeping 3 PA low. Pumping at longer wavelengths to get rid of 3 PA, or using shorter input pulses to mitigate the free-carrier penalty represent some solutions that could make the most of this platform, and eventually lead to SCG potentially covering the whole MIR region. The main results reported in Ge and SiGe based devices are summarized in Table 5.

**Table 5:** Summary of the main results presented in Ge and SiGe based devices.

Platform	Width × height, length	Pump(nm)	*γ* (mW)^−1^	*α* (dB/cm)	Coupling loss	$P_{\text{P}}^{\text{C}}$ (W) ( $P_{\text{A}}^{\text{C}}$ (mW))	Bandwidth	Year
Graded index SiGe [86]	1.22 μm × 1.597 μm, 20 mm	2400	24.7	2	10.5 dB/facet	1200 (0.94)	1.45–2.79 μm	2015
SiGe-on-Si [22]	2.7 μm × 3.75 μm, 70 mm	4000	0.63	0.35–0.5	4.9 dB/facet	2350 (16)	2.63–6.18 μm	2018
SiGe-on-Si [22]	4.2 μm × 6 μm, 70 mm	4150	0.3	0.23 (at 4.75 μm)	4.9 dB/facet	2540 (25)	3–8.3 μm	2018
SiGe-on-Si, chalcogenide cladding [87]	2.7 μm × 3.75 μm, 50 mm	4150	0.57	0.55	4.9 dB/facet	2320 (15.8)	3.1–5.5 μm	2019
SiGe-on-Si [15]	2.7 μm × 5 μm, 20 mm	4000	0.72	0.3	4.2 dB/facet	5720 (39)	2.8–5.7 μm	2020
Graded index SiGe [59]	6.1 μm × 6.2 μm, 5.5 mm	8500	0.4	0.5–1.2	7.2 dB/facet	2600 (0.32)	3–13 μm	2020
Ge-on-Si [25]	2.57 μm × 4.46 μm, 22 mm	4600	1.59 (TE), 1.65 (TM)	1.2–1.35	6.8 dB/facet	3300 (22) (TE), 4500 (30) (TM)	3.53–5.83 μm (TE), 3.33–5.53 μm (TM)	2021
SiGe-on-Si [88]	3.3 μm × 3.25–9 μm (tapered), 23 mm	3900	0.37–1	0.12–0.7	5.3 dB/facet	2730 (18.2)	2.4–5.5 μm	2022

Eventually, whether it be for covering the visible, near-IR, or longer MIR band, Si-based waveguides have proved to provide several viable routes for achieving efficient chip-based SCG devices. While initially leveraging the Si photonics manufacturing processes, specific fabrication strategies have been further developed for the fabrication of these devices, offering today some of the most mature platforms for implementing SCG. In parallel to these continued efforts devoted on Group IV materials, other integrated materials have yet been investigated, as they offer either superior intrinsic nonlinear characteristics or complementary nonlinear properties (combined *χ* ^(2)^ and *χ* ^(3)^ for instance) that could be leveraged for SCG. We review these alternative platforms in the next sections.

## 4 Supercontinuum generation in chalcogenide waveguides

Chalcogenide (ChG) glasses are amorphous semiconductors. They contain one or more chalcogen elements (sulfur, selenium, and tellurium) as major constituents, which are covalently bonded to a network of atoms such as As, Ge, Sb, Ga, Si, or P. Chalcogenides have unique electrical and optical properties, and they have been widely studied for electronic and photonic applications [41]. In the context of reaching broadband SCG, their main advantage is the wide transparency window that goes from the near-IR to beyond 11 μm and up to 20 μm, depending on their material composition (see Table 1). Nonlinear waveguides made of specific ChG compounds such as Ge_11.5_As_24_Se_64.5_ and As_2_S_3_ also benefit from relatively high *γ* (0.1–10 m^−1^ W^−1^), negligible TPA, even at telecom wavelengths and, consequently, no resulting free-carrier penalty [41].

**Figure 13:**
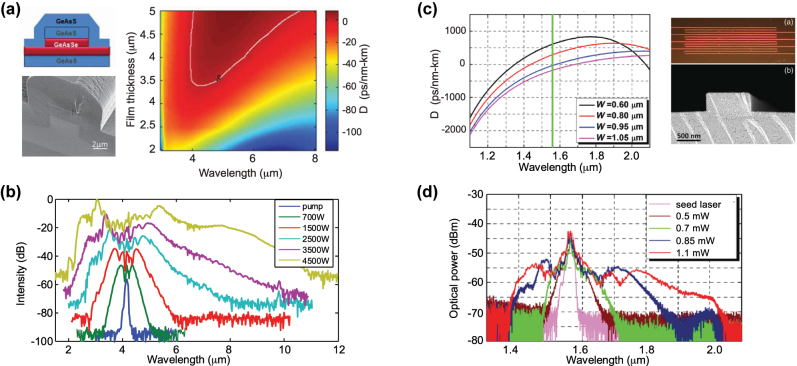
SCG in chalcogenide waveguides. (a) Schematic (top) and SEM image (bottom) of the Ge_11.5_As_24_Se_64.5_ buried-in Ge_11.5_As_24_S_64.5_ waveguide, and calculated dispersion as a function of the wavelength and core film thickness for the fundamental TM at an etch depth of 50% and a fixed waveguide width of 4 μm. (b) Experimental SC at the waveguide output for different pump powers for TM mode. (c) Simulated dispersion of GeSbSe waveguides with varying widths and a fixed core thickness of 0.4 μm, and top-view optical microscope image and SEM cross-section image of the GeSbSe waveguide. (d) SC generation of a 21 mm long GeSbSe waveguide with varying average input power. Panels (a) and (b) adapted from [60]. Panels (c) and (d) adapted from [118].

The first demonstration of SCG in a ChG waveguide by Psaila et al. dates back to 2007, with the generation of an SC spanning from 1320 to 1920 nm in a laser-inscribed waveguide [112]. This kind of waveguide, however, typically presents low index contrast and a large effective area (the waveguide had an elongated cross-section of around 40 μm × 100 μm), yielding a low nonlinear parameter, hence relatively inefficient SCG. The following demonstrations have then been reported in rib and ridge waveguides, which allow for tighter mode confinement. A review of the most common fabrication methods can be found in [41]. Typically, ChG films are grown by thermal evaporation [113], sputtering [114], chemical vapor deposition [115], or pulsed laser deposition [116], and the final waveguide is created by structuring the film through lithography and etching. In addition, strong efforts have been deployed to provide specific ChG compounds (such as Ge_11.5_As_24_Se_64.5_ [117]) that exhibited an optimized trade-off between well-suited nonlinear properties for chip-based applications and high material stability, which tends to otherwise impede the performance of ChG nonlinear devices. The improvement in the fabrication techniques in the last fifteen years thus led to the demonstration of low propagation loss waveguides (0.03–0.5 dB/cm, see Table 6) and SCG both in the near-IR [118–121] and in the MIR [60, 117, 122, 123]. Notably, Yu et al. reported in 2016 SCG from 2.2 to 10.2 μm in a Ge_11.5_As_24_Se_64.5_ core rib waveguide with Ge_11.5_As_24_S_64.5_ upper and lower cladding (Figure 13(a) and (b)) operating in TM polarization [60]. This was the first result in any integrated platform that could compete with fibers in terms of optical bandwidth of the generated SC. As compared to previous results [117, 122], the symmetric nature of the waveguide (upper and lower cladding of the same material) enabled to extend the cutoff to beyond 10 μm, thereby widening the bandwidth of the generated SC. Interestingly, in TE polarization the waveguide operated in the ANDi regime, and a relatively narrow SC extending from 2.4 to 6.5 μm was reported. The waveguide also showed extremely low polarization mode coupling, which allowed performing dual beam spectrophotometry of polystyrene even though the SC spectra had intensity fluctuations in time. Another interesting example is the generation of SC in GeSbSe zig-zag waveguides pumped at telecom wavelength [118] and illustrated in Figure 13(c) and (d). Leveraging the uncladded design, they could use waveguides of different lengths to quantify the CH bond overtone absorption of chloroform at around 1.7 μm wavelength.

**Table 6:** Summary of the main SCG results presented in chalcogenide based waveguides.

Platform	Width × height, length	Pump(nm)	*γ* (mW)^−1^	*α* (dB/cm)	Coupling loss	$P_{\text{P}}^{\text{C}}$ (W) ( $P_{\text{A}}^{\text{C}}$ (mW))	Bandwidth	Year
As_2_S_3_-on-SiO_2_ [119]	0.87 μm × 2 μm, 60 mm	1550	10	0.6	3.7 dB/facet	68 (0.6)	1.15–1.7 μm	2008
Ge_22_Sb_18_Se_60_-on-SiO_2_ [118]	0.4 μm × 0.95 μm, 21 mm	1560	N.A.	4	7 dB/facet	170 (1.1)	1.4–2.05 μm	2018
Ge_23_Sb_7_S_70_-on-SiO_2_ [120]	0.91 μm × 1.2 μm, 20 mm	1550	N.A.	0.56	7 dB/facet	106 (0.64)	1.03–2.08 μm	2018
As_2_S_3_-on-SiO_2_ [121]	Trapezoidal waveguide, 1.05 μm^2^ eff. area, 10 mm	1560	11.1	0.03	10 dB/facet	502 (7.02)	1.05–2.71 μm	2021
As_2_S_3_-on-SiO_2_ [117]	2.5 μm × 4 μm, 66 mm	3260	0.45	0.75	5 dB/facet	1700 (15.8)	2.9–4.2 μm	2012
Ge_11.5_As_24_Se_64.5_-on-Ge_11.5_As_24_S_64.5_ [122]	2.5 μm × 4 μm, 10 mm	4000	0.49	0.5	5.6 dB/facet	1640 (80)	1.8–7.5 μm	2014
Ge_11.5_As_24_Se_64.5_-on-Ge_11.5_As_24_S_64.5_ [60]	4.4 μm × 4 μm, 18 mm	4184	0.2	0.6	6 dB/facet	4500 (32.7)	2.2–10.2 μm	2016
As_2_S_3_-on-SiO_2_ [123]	Nanospike waveguide 5.4–3 μm core, 4.5 mm	2800	0.15–0.4	0.01–10 between 1 and 5 μm	13.77 dB/facet	4200 (14.5)	1.1–4.8 μm	2021

These demonstrations showed that ChG waveguides are well suited for chip-based ultra-broadband SCG. Their main drawback still tends to be the relatively high material photosensitivity [41]. Although this has been proved useful as a tool for creating photo-written high-*Q* cavities [132], or achieving device reconfigurability [133], this property can translate for some ChG compounds into low temporal stability of the SC source and material damaging at high optical powers. In addition, despite their record high nonlinear performance, it remains hard for ChG waveguides to compete with group IV (Si, SiN, SiGe, and Ge) platforms that advantageously offer more straightforward solutions for large-scale, low-cost fabrication of integrated optical chips.

## 5 Supercontinuum generation in *χ* ^(2)^ – *χ* ^(3)^ waveguides

**Figure 14:**
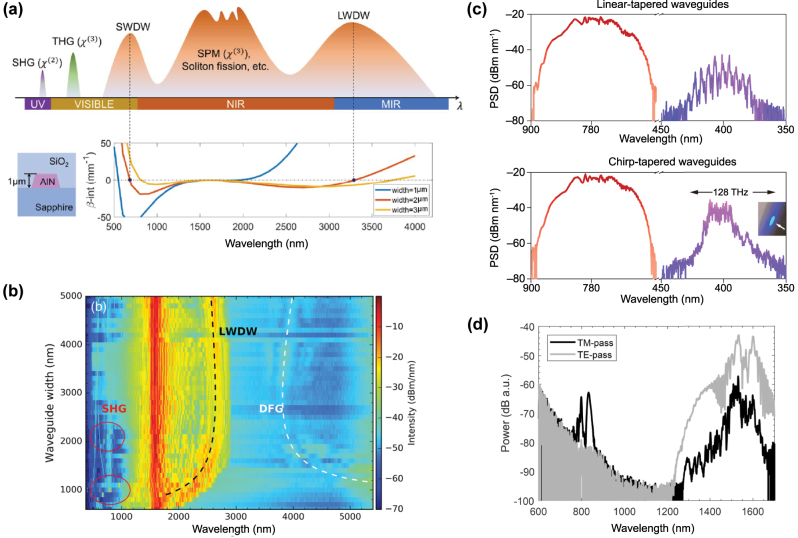
Simultaneous SCG and three wave mixing processes in waveguides based on semiconductor alloys. (a) Schematic representation of a UV-to-MIR spectral spanning including SCG, SHG, and THG in an AlN waveguide. (b) Experimental spectra showing combined SCG, SHG, and DFG within the same AlN waveguide. The spectral position of the different processes can be tuned by changing their phase-matching condition by changing the width of the waveguide. (c) Example of highly coherent ANDi SCG and broadband frequency conversion towards UV via SHG in a chirp-modulated tapered AlN waveguide. (d) TE and TM components of the output spectra from a suspended AlGaAs waveguide pumped by 61 fs pulses at 160 MHz at 1560 nm in TE polarization. The SCG is mostly generated with same polarization of the pump while SHG is generated in TM polarization, as expected for waveguides along the [011] axis. Panel (a) adapted from [33]. Panel (b) adapted from [35]. Panel (c) adapted from [61]. Panel (d) adapted from [30].

SC generation in materials exhibiting both *χ* ^(2)^ and *χ* ^(3)^ type of optical nonlinearities has been increasingly investigated driven by the potential to build an f-to-2f interferometer (see Section 7.1) out of just one nanophotonic waveguide. The main focus has been on realizing devices that can accept pump light in the telecom wavelength range, a region where most of the femtosecond fiber-lasers are developed but where the technologically very mature silicon photonic devices are limited by TPA, as discussed in Section 3.1. Such application requires a high third order nonlinearity to reach an octave-spanning SC, a sufficiently large band gap to reduce nonlinear losses at the pump wavelength and high second-order nonlinearity for efficient SHG. However, more recently, the combination of *χ* ^(2)^ and *χ* ^(3)^ properties within the same waveguide is also pursued to achieve a spectral extension beyond what is normally possible by SCG only, as stimulated three-wave mixing processes like SHG, sum frequency generation (SFG) and difference frequency generation (DFG) guarantee a coherent frequency conversion (Figure 14(a)). These effects become particularly powerful when combined with efficient phase matching using the birefringence of the materials or quasi phase matching via periodic poling, which is possible in several material platforms. Moreover, cascaded *χ* ^(2)^-based process can act as an effective *χ* ^(3)^ process much larger than the intrinsic *χ* ^(3)^ of such device, leading to very efficient SPM and SCG. Indeed, it can be shown [134] that in the case of phase mismatched SHG (Δ*β* > 0) and for $\left|\Delta \beta \right|\gg 4\pi \left|\Delta \beta^{\prime }/\tau \right|$ , with Δ*β*′ > 0 being the group velocity mismatch between the pump and second harmonic and *τ* the transform-limited pump pulse duration, the effective third order nonlinearity scales as 1/Δ*β*. Therefore, the possibility to dispersion engineering integrated waveguides to reduce Δ*β*′ allows for a great enhancement of the spectral broadening. The most promising candidates experimentally tested so far are semiconductors alloys and LNOI waveguides. We review them in the next two sections.

### 5.1 Devices based on material alloys

Material alloys made of group III and group V elements of the periodic table naturally exhibit a non-centrosymmetric crystalline structure and therefore a *χ* ^(2)^ optical nonlinearity. Among the different possible compounds, SCG has been reported in InGaP, AlGaAs, and AlN. In particular, AlN offers the largest available band gap (≃6.2 eV), a refractive index of about 2.12 for the ordinary axis, and a nonlinear refractive index *n*_2_ ≃ 2.3 × 10^−15^ cm^2^/W at 1550 nm, values similar to Si_3_N_4 _(see Table 1). From 2012, nanophotonic structures based on AlN have been fabricated from sputtered AlN thin films on top of thermally grown SiO_2_, leading to waveguides with low propagation losses in the telecom range while maintaining low deposition temperatures compatible with CMOS processes [32]. In such devices, AlN is in a poly-crystalline arrangement in which the *z* axis of the crystal, which has the largest *χ* ^(2)^ tensor element (*d*_33_ = 3 pm/V), is perpendicular to the plane of the wafer for all the domains, while the other axis are randomly oriented. Therefore, the TM polarization makes full use of the large second-order nonlinearity and the waveguides should be dispersion engineered to achieve SCG for the TM mode. In 2017, Hickstein et al. [35] demonstrated accurate dispersion engineering in fully SiO_2_-clad AlN-on-insulator waveguides by varying the waveguide width from 400 to 5100 nm, achieving SCG from 1400 nm to 2900 nm at −20 dB in the fundamental TE mode. An even broader spectrum is likely to be limited in the long wavelength region by the strong absorption of the weakly confined MIR mode caused by OH bonds in the silica cladding above 2900 nm. When pumped in the TM fundamental mode, the SCG spans the range 1400–2700 nm at −20 dB, however the optical spectrum is extended at short wavelength by SHG and beyond 3500 nm by DFG between the broadened pump and the DW in the red part of the spectrum (Figure 14(b)). Despite the limited SC power in the NIR/VIS available in the TM mode, the authors could demonstrate *f*_CEO_ detection by simultaneous SCG and SHG.

Another strategy to fabricate AlN waveguides consists in epitaxially growing single-crystalline AlN thin films on sapphire. This approach promises low propagation losses both at MIR, thanks to the transparency of the sapphire substrate, and in the VIS/UV range, thanks to the reduced scattering losses of the single-crystal structure [103]. Indeed, in this platform Liu et al. have shown coherent frequency comb generation in the UV by SHG of a near-IR/VIS comb obtained by an all-normal-dispersion (ANDi) SCG pumped at 780 nm [61] (Figure 14(c)). In this case, the authors leveraged on the spectral flatness and the high degree of coherence of the ANDi SCG process in conjunction with a chirp-modulated tapered waveguide to obtain a spectrally flat UV comb spanning 128 THz around 390 nm. Ultraviolet to MIR SCG has been obtained in similar waveguides. In particular, the authors showed tunable VIS and MIR (up to 3500 nm) DWG by pumping the TE fundamental mode at telecom wavelength and UV generation by cascaded SHG on higher order TM modes when the sample is pumped in the TM fundamental mode.

In contrast to waveguides made of the insulator AlN, nanophotonic waveguides based on other III-V semiconductors combine a large refractive index of the core (*n* > 3), with very high optical nonlinearities. In particular, the *n*_2_ is similar to or higher than that of Si while the second order nonlinear coefficient (*d*_14_ = 80–120 pm/V) is much larger than that of AlN. Also, their bandgap can be large enough to avoid TPA at telecommunication wavelengths. Their main limiting factor has been the difficulty of fabricating large index contrast waveguides. In fact, the most commonly used III–V compounds such as InGaP or Al_*x*_Ga_1−*x*_As are epitaxially grown on a GaAs substrate with a high refractive index of *n* = 2.86 leading to a weak index contrast (Δ*n* ≃ 0.2) and poorly confined modes, which result in low nonlinear coefficients as well as limited possibilities for dispersion engineering and dense integration. Alternatively, suspended waveguides or thermally oxide layers have been investigated to achieve large index contrast waveguides at the expenses of higher propagation losses [135, 136]. Only recently, heterogeneous integration of III–V semiconductors on SiO_2_ substrates via wafer bonding has proven to be a reliable way to exploit the benefits of such alloys in loss competitive platforms [29, 37, 38, 137, 138].

The first SCG in such III–V semiconductor waveguide was demonstrated by Dave et al. in 2015 [38] using InGaP on insulator. Notably, the authors achieved an octave of bandwidth through DW generation, pumping the waveguide with a 1550 nm femtosecond laser. Besides InGaP, Al_*x*_Ga_1−*x*_As has been investigated even earlier for nonlinear applications. The band gap of such compound has the advantage that it can be tuned by changing its Al content, also reducing nonlinear losses. Octave spanning SCG has been achieved in AlGaAs-on-insulator by Kuyken et al. in 2020 [28] leveraging soliton compression and DW generation in a waveguide just 3 mm long. Such dynamics have been confirmed by systematic studies of SCG by May et al. in 2021 [126]. In 2019, Chiles and co-authors demonstrated a multifunctional photonic integrated circuit (PIC) in AlGaAs bonded on a Si substrate [30]. The fabrication process was akin to the one used for heterogeneous integration on oxide layers, however here the host material is an etched Si substrate which allowed for the fabrication of suspended waveguides, and hence long wavelength operation. Indeed, the authors demonstrated both an octave spanning SC pumped at telecom wavelength and an MIR pumped SCG up to 6.5 μm. The waveguides along the [011] axis also exhibited SHG in TM polarization when pumped at 1560 nm in TE polarization (Figure 14(d)).

Investigation of a promising and closely related wide band-gap III–V semiconductor is currently pursued on GaP materials that exhibit both a high *χ* ^(2)^ and *χ* ^(3)^ nonlinearity, no TPA, and an even broader transparency range (0.55–11 μm) than InGaP. Although an actual SCG has not been demonstrated yet, interesting and relevant developments of efficient nonlinear devices in the GaP on insulator platform have been recently reported [139], which open up new prospects for broadband chip-based SCG.

### 5.2 LNOI based devices

Similar to the III–V materials, lithium niobate (LN) is a versatile optical material which has played an important role in optics for many years. Its outstanding properties are the comparably large *χ* ^(2)^ nonlinearity and electro-optic coefficient. The *χ* ^(2)^ nonlinearity combined with the ability to pole, which means to locally change the sign of the *χ* ^(2)^ nonlinearity along the extraordinary axis, has made LN one of the most commonly used nonlinear materials, especially at telecom wavelengths. It is used in applications such as frequency doubling of lasers, optical parametric oscillators, self-referencing of frequency combs, and difference frequency generation. Furthermore, with its high electro-optic coefficient, LN is the standard material for high-speed electro-optic modulators as they are used in datacom and many other applications.

In many of these applications, LN has been used in some form of waveguide for several tens of years now [140]. These waveguides have been made using different technology such as proton exchange, ion diffusion, or blade dicing. However, none of these technologies enabled the small waveguide cross sections with high confinement and the fine control of the waveguide dimensions which are required for efficient SCG. The ability to make nanophotonic waveguides only emerged in 2007 [141] and depended crucially on the development of the thin film LNOI platforms and their broader availability. The process to make LNOI wafers is closely related to the crystal ion slicing (CIS or also known as Smart Cut) technology, which was originally developed in 1994 to make silicon on insulator (SOI) wafers. This process makes the transfer of mono-crystalline thin films onto amorphous silicon dioxide and other substrates possible. The invention of Smart Cut has enabled silicon photonics to take off. Similarly, the availability of commercial LNOI wafers was also the prerequisite for the widespread development of LN integrated photonics. The first reports of CIS LNOI chips were published in 2007 [141] and reports on the wafer-scale followed soon after [142]. A few years later, this promising material stack became commercially available. Another important step forward was the fabrication of low-loss waveguides using such substrates for different wavelength ranges [143, 144]. Since then, LNOI has been used to implement a very diverse set of optical functionalities on PICs and represents a very promising platform [42, 145].

**Figure 15:**
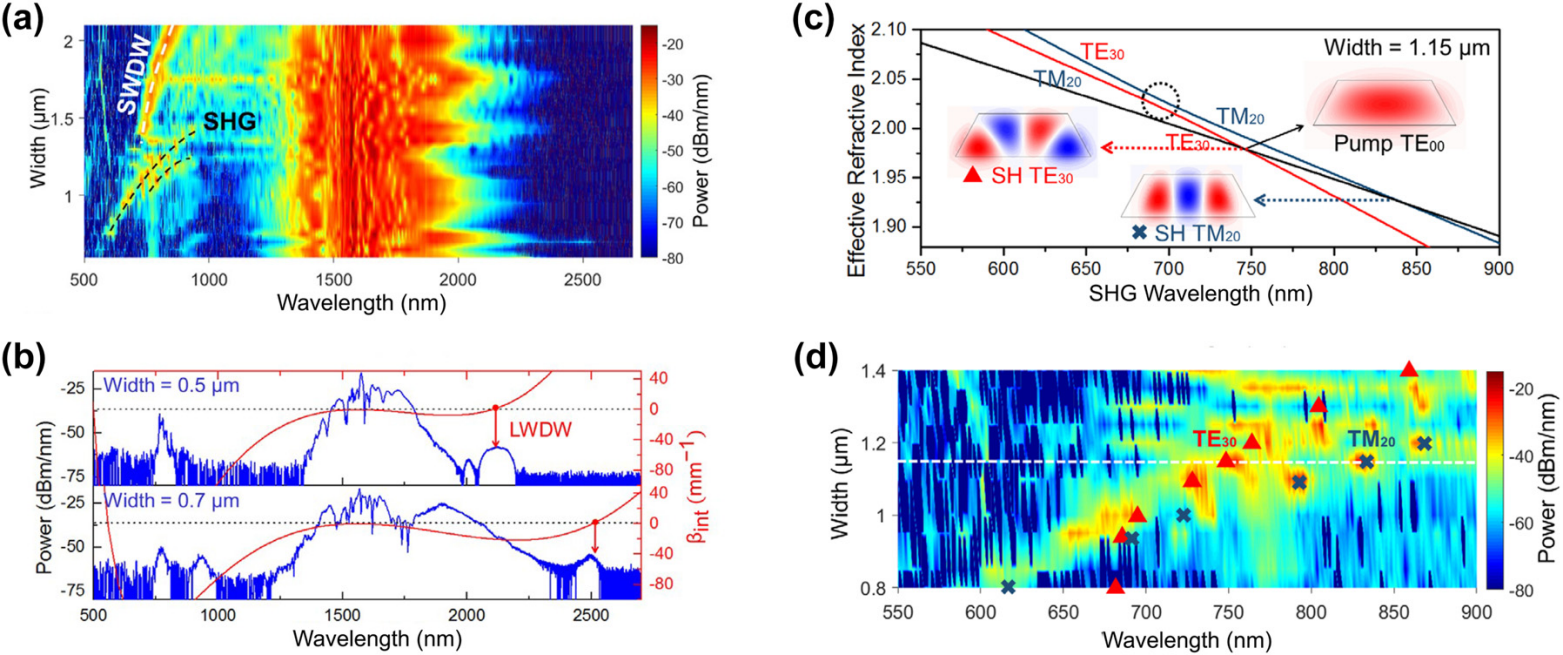
SCG in LNOI waveguides. (a) Experimental spectra compiled for many different waveguide widths. (b) Experimental spectra (blue) including simulated integrated dispersion (red) for two waveguide widths. (c) Effective refractive index for several modes for a waveguide width of 1.15 um. (c) Simulated phase-matching wavelengths for higher order modes (TE30 as red triangles and TM20 as blue crosses) mapped onto experimental data for different waveguide widths. Adapted from [127].

**Figure 16:**
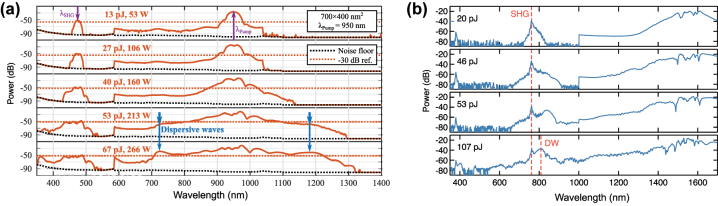
Evolution of SCG in LNOI waveguides for different pulse energies. (a) A waveguide with a top width of 700 nm and a height of 400 nm pumped at 950 nm. (b) A waveguide with a top width of 1250 nm and a height of 800 nm pumped at around 1560 nm. Panels (b) and (a) adapted from [129, 130] respectively.

The first published results of SCG in nanophotonic LNOI waveguides were reported in 2019 [127] and are shown in Figure 15. The work also included an analysis of the role of phase matching with higher order modes in the generation of the second harmonic light, which is clearly visible as a peak at half the pump wavelength. First results including the measurement of the self-referencing signal of a femtosecond laser as an f-to-2f beatnote followed [128, 129, 134]. The generation of the SC and the SH in the same LNOI waveguide (Figure 16(b)) does allow for a very simple and direct detection of the self-referencing beatnote with conventional photodiodes close to the end of the waveguide on the chip. Despite the comparably low input powers, the compact size, and the simple detection, this configuration can achieve signal to noise ratios of beyond 50 dB and, as a consequence, these PICs can realize very stable self-referenced frequency combs [62]. Other demonstrations focused on the extension of the SC towards the visible and blue by exploiting the second-order nonlinearity to double the pump light frequency [130]. In that work, the waveguide is pumped with light at around 940 nm. A part of the pump light is doubled in frequency, initiating an SCG process around 470 nm, which finally reaches down to 352 nm (Figure 16(a)). While the resulting spectrum covers nearly two octaves, it should be highlighted again that, as mentioned before, the spectrum does most likely not represent a single, coherent frequency comb due to different offset frequencies in the different parts of the spectrum generated via SCG and HG.

Both of these applications, the self-referencing and the generation of broadband visible and blue light could benefit from periodic poling of the waveguide. Several demonstrations of periodic poling of LNOI have been published and, for SHG, it has resulted in very high conversion efficiencies [146, 147]. A first demonstration of SCG in a periodically poled nanophotonic dispersion engineered LNOI waveguide has been published in 2020 as well [134]. It features an impressive demonstration of the power of the cascaded second-order interactions compared to the usual third-order interaction used in SCG. The authors claim an effective third-order nonlinearity which is a factor of 200 higher compared to the intrinsic third-order nonlinearity of LN. One promising route towards even more powerful SCG could be the adaption of more sophisticated techniques, which optimize the dispersion and the phase matching to obtain broadband matching as reported for nanophotonic optical parametric amplifiers [148, 149].

In contrast to some of the other platforms described here, the waveguides in LNOI are often not fully etched. Typical dimensions are a waveguide height of 600 nm of which only around 300–400 nm are etched. Therefore, there is a substantial LN slab remaining, which affects the dispersion, mode shape, and losses of the waveguide. In particular, for the dispersion engineering and the input and output coupling this slap has to be taken into account.

While LNOI is certainly a very promising material for integrated photonics in general and SCG in particular, there are a few known issues with this material platform today. The first one is the photo-refractive effect. This effect is already well known from bulk LN crystals [150] and it does play a role for LNOI as well. How much it limits the optical power that LNOI waveguides can handle still has to be determined. But in LNOI microresonators, very clear effects attributed to the photo-refractive effect have been observed [151]. Countermeasures include the use of doped LN and it is generally understood that impurities play a large role. The latter are a problem as the growth of LN is much less mature than for e.g. silicon and on top of this LN is a more complicated crystal to grow, with a difference between congruent crystal and a stoichiometric one. The usual LN crystals are congruent, which means that the crystal cannot be perfect, in contrast to stoichiometric crystals. For the SC waveguides there have been reports on damage at the input facet [62, 130], but the reason for this is still not very well studied. Simple solutions to this problem could be an appropriate top cladding and a better design of the input section. Despite this, the combination of a maturing PIC platform, its ability of quasi-phase matching via periodic poling, and waveguide dispersion engineering offer significant opportunities for nonlinear integrated optics in general, and SCG in particular. The main SCG results presented in waveguides with both second and third order nonlinearity are summarized in Table 7.

**Table 7:** Summary of the main SCG results presented in *χ* ^(2)^/*χ* ^(3)^ devices.

Platform	Width × height, length	Pump (nm)	*γ* (mW)^−1^	*α* (dB/cm)	Coupling loss	$P_{\text{P}}^{\text{C}}$ (W) ( $P_{\text{A}}^{\text{C}}$ (mW))	Bandwidth	Year
InGaP/SiO_2_ [124]	0.7 μm × 0.25 μm, 2 mm	1550	450	12	27 dB/facet	10 (0.14)	1–2.097 μm	2015
*p*-AlN/SiO_2_ [35]	0.4–5.1 μm × 0.8 μm, 10 mm	1560	N.A.	N.A.	4 dB/facet	4000 (80)	0.51–4 μm	2017
c-AlN/Sapphire [61]	0.42 μm × 0.5 μm, 6 mm	780	9.5	> 6	4 dB/facet	2370 (19)	0.6–1.05 μm	2019
Suspended AlGaAs [30]	0.48 μm × 0.53 μm, 4 mm	1560	165	0.54	4.5 dB/facet	56 (0.5)	1.15–2.3 μm	2019
Suspended AlGaAs [30]	2.15 μm × 0.53 μm, 2.3–10.5 mm	3060	30	0.54	4.5 dB/facet	530 (4.5)	2.3–6.5 μm	2019
AlGaAs/SiO_2_ [28]	0.5 μm × 0.3 μm, 3 mm	1555	630	2	12 dB/facet	97 (0.873)	1.055–2.155 μm	2020
c-AlN/Sapphire [33]	2.6 μm × 1 μm, 8 mm	1560	0.47	0.45	5 dB/facet	3500 (56)	0.35–3.5 μm	2020
c-AlN/Sapphire [125]	1.1 μm × 1.2 μm, 6 mm	810	N.A.	20	8.2 dB/facet	3600 (30)	0.49–1.1 μm	2021
AlGaAs/SiO_2_ [126]	0.7 μm × 0.27 μm, 3 mm	1560	N.A.	2–3	6 dB/facet	31 (0.248)	1.3–1.9 μm	2021
LNOI/SiO_2_ [127]	0.5 μm × 0.6 μm–3 μm × 0.8 μm, 10 mm	1560	0.4	0.16	6.5 dB/facet	4000 (64)	0.75–2.25 μm	2019
LNOI/SiO_2_ [128]	0.8 μm × 0.8 μm to 2.3 μm × 0.8 μm, 5 mm	1506	N.A.	3	8.5 dB/facet	1170 (15)	0.4–2.400 μm	2019
LNOI/SiO_2_ [129]	1.25 μm × 0.8 μm, 5 mm	1560	N.A.	N.A.	10.3 dB/facet	1200 (26)	0.58–>1.7 μm	2020
LNOI/SiO_2_ [130]	0.7 μm × 0.4 μm, 14 mm	950	N.A.	N.A.	11.2 dB/facet	266 (5.4)	0.35–1.35 μm	2020
LNOI/SiO_2_ [62]	1.3 μm × 0.6 μm, 5 mm	1555	N.A.	N.A.	6.2 dB/facet	950 (14)	0.66–>2 μm	2021
LN/SiN/SiO_2_ [131]	N.A., 10 mm	1560	N.A.	0.085	5 dB/facet	3400 (31)	0.95–>1.7 μm	2021

## 6 Supercontinuum generation in other integrated platforms

In recent years, several other material platforms have been suggested as potential interesting alternatives for SCG, although at this time there are only a handful of demonstrations and they remain less technologically mature. We summarize in Table 8 the demonstrations of SCG in these other platforms, namely in diamond, silicon carbide (SiC), tellurium dioxide (TeO_2_), tantalum pentoxide (Ta_2_O_5_), and titanium dioxide (TiO_2_).

**Table 8:** Summary of the main SCG results presented in section 6.

Platform	Width × height, length	Pump (nm)	*γ* (mW)^−1^	*α* (dB/cm)	Coupling loss	$P_{\text{P}}^{\text{C}}$ (W) ( $P_{\text{A}}^{\text{C}}$ (mW))	Bandwidth	Year
Diamond [152]	0.54 μm (100° apex), 5 mm	810	N.A.	1.5	10 dB/facet	210 (1.7)	0.67–0.92 μm	2019
SiC [153]	1.065 μm × 0.5 μm, 9 mm	1558	N.A.	N.A.	3.5 dB/facet	75 (40)	1.3–1.7 μm	2020
TeO_2_ [154]	0.37 μm on top of 1.2 μm × 0.2 μm SiN, 7 mm	1550	N.A.	0.5	15 dB/facet	600 (9.6)	1.35–1.85 μm	2020
Ta_2_O_5_ [155]	0.8 μm × 0.7 μm, 5 mm	1056	34.2	1.5	18 dB/facet	396 (3)	0.58–1.697 μm	2019
Ta_2_O_5_ [156]	2.6 μm × 0.7 μm, 18 mm	1000	N.A.	3	10 dB/facet	∼1400 (17.5)	0.73–1.3 μm	2020
TiO_2_ [157]	1.4 μm × 0.45 μm, 22 mm	1640	1.2	5.5	11 dB/facet	1300 (9.4)	1.05–1.91 μm	2018

Diamond combines a wide bandgap, a high refractive index as well as good optical nonlinearity. It is a platform already widely exploited for quantum applications since it hosts optically active color centers, but it is only emerging for integrated nonlinear photonics [47]. It was shown that the structure can be engineered as to reach anomalous dispersion at the pump wavelength of interest, such as the telecom or visible band [158]. Diamond seems indeed well suited for visible SCG. It was shown in [159] that we can reach a ZDW in the visible based on a diamond-on-insulator (DOI) design. The simulations indicated that from a 720 nm input pump with 230 W peak power, an SC spanning 0.453 μm–1.03 μm could be obtained in a 4 mm long waveguide. The only experimental demonstration so far uses angle-etching of diamond rather than DOI. One of the difficulties with DOI is the low device yield as well as some thickness fluctuations of the fabricated devices, which can be detrimental for the dispersion engineering of nonlinear devices. The alternative to avoid such fluctuation is to angle-etch bulk diamond, which results in free-standing structures, which also have been used for quantum photonics and opto-mechanics [160, 161]. In waveguides with 540 nm width and 100° apex angle, a pump at 810 nm resulted in an SC spanning from 0.67 μm to 0.92 μm [152]. The performance was limited by sidewall roughness and the coupling facet, which degraded at high pump powers, resulting in a graphitization phenomenon which extended along the waveguide. Improvement in the waveguide and coupler design are the next steps in order to allow operation with lower pulse energies to avoid any damage and extend the SC further towards the UV.

Silicon carbide is another material platform that is attracting a lot of attention from the quantum technology but also the nonlinear photonic community. While SiC is currently less established in the PIC area, this wide bandgap compound, CMOS-compatible semiconductor combines a large optical transparency up to the MIR, high second and third order nonlinearities, and the crystalline form can host quantum emitters with controllable electronic spins due to the availability of different point defects. The amorphous and crystalline forms of SiC have been studied. While amorphous SiC can be directly deposited by PECVD on a lower index material, hence facilitating fabrication, it shows compromised mechanical properties, lower nonlinearities and some three-photon absorption [162]. Eventhough some SPM was observed in such a-SiC waveguide, the broadening was extremely limited. Over the 200 polytypes of SiC, the most common and advanced crystalline forms used for integrated optics are the hexagonal 4H-SiC and 6H-SiC as well as the common cubic form 3C-SiC. The latter is available as an epitaxially grown layer on Si, but creates a challenge for the design of waveguides since the 3C-SiC (index *n* = 2.6) needs to be optically isolated from the underlying Si which has a higher index. The structures have to be suspended. In addition, the intrinsic loss of 3C-SiC is relatively high due to electrically active stacking defects from the lattice mismatch between SiC and Si [163]. Significant work has therefore been focused on the use of 4H-SiC. Both 4H and 6H-SiC are only available at the moment as bulk materials but with the highest purity and crystal quality. The required thin SiC film with a low-index cladding thus requires processing of the bulk material without compromising the quality. To that end, the Smart Cut technique [164] or a wafer bonding and thinning technique [165] have been used. While the latter resulted in better quality waveguides and higher-*Q* microresonators, up to now the only spectral broadening beyond SPM has been demonstrated with a Smart Cut fabrication of 4H-SiC-on-insulator waveguides pumped at telecom wavelength [153]. While SPM broadening of only 97 nm was obtained for a narrow waveguide exhibiting normal dispersion at the pump wavelength, the waveguide with a 1065 nm width exhibited anomalous dispersion such that a spectrum spanning 1.3–1.7 μm could be generated. This first demonstration is encouraging, and further dispersion engineering and optimization are necessary to push the performance of the platform.

An SCG generated in TeO_2_ was presented in [154]. The interest in exploiting the nonlinearity of TeO_2_, with a relatively high nonlinear refractive index estimated close to 40 times that of silica, is in the possibility to combine these functionalities with active properties. Tellurite glasses can host various rare-earth ions for use in sources and amplifiers [166]. However, difficulties in the fabrication of integrated structures with low loss and high confinement have limited the potential of the platform for nonlinear optics. In [154], TeO_2_ is used as a thin 370 nm layer on top of a SiN waveguide core. It is estimated that 70% of the total mode energy is confined in the TeO_2_. The dispersion of the structure, however, was normal and relatively high, such that only 500 mn of broadening could be obtained around the 1550 nm pump. Simulations show that the reach could be extended by optimizing the dimension of the SiN core to reach anomalous dispersion at telecom wavelength and 2 ZDWs for possible DW generation.

Similarly, Ta_2_O_5_ is another CMOS-fabrication compatible material that can also host rare-earth ions showing potential for lasing while exhibiting a high nonlinear refractive index. A wide SC spanning 1.5 octaves could be generated in a Ta_2_O_5_ waveguide, air-cladded as to reach the desirable anomalous dispersion around 1 μm [155], with relatively low input pump power, showing the potential of the platform. In [156], the authors performed a detailed study of the behavior of the spectral broadening in both air-clad and silica clad Ta_2_O_5_ waveguides with large cross sections. They studied the influence of the pump wavelength and polarization state. The waveguides under test having a large cross-sections, it is shown that multimode behavior when pumping at low wavelengths can be relatively well modelled and leveraged to reach desirable dispersion regimes.

A last alternative platform considered for SCG is TiO_2_, which shows very similar properties to the latter two oxide materials. Still, little work has been done with this material in terms of nonlinear optics, with one main SCG demonstration reported in [157]. The waveguides were fabricated by depositing a 450 nm thick layer of TiO_2_ by DC magnetron sputtering, resulting in a polycrystalline anatase phase. Waveguides with either rutile phase [167] or amorphous [168] can also be fabricated. While amorphous TiO_2_ shows lower propagation losses, anatase waveguides have a higher reported nonlinearity and require lower fabrication temperatures than crystalline rutile waveguides. A soliton-based SCG from 1640 nm input pulses is obtained, reaching a spectral coverage from the visible up to 1.92 μm. The propagation losses are however still relatively high and it is speculated that using TiO_2_ in amorphous phase, together with improvement in dispersion engineering, could push the performance of this platform. Overall, TiO_2_ but also TeO_2_ and Ta_2_O_5_, exhibit a wide transparency window, easily reaching the MIR (between 5 and 8 μm) such that optimization of the fabrication and control of the dispersion holds a high potential for significantly extending the reach and efficiency of the SCG, as has been done in more mature chip-based platforms such as Si_3_N_4_.

## 7 Applications of chip-based supercontinuum

Following these state-of-the art reviews of SCG demonstrated in various integrated platforms, which highlighted, for each of them, the currently achieved performance level as well as the technological maturity and remaining challenges, we next attempt to draw an overview of the main applications opened up by SCG. Indeed, the common and intrinsic features of SC, whether it be their temporal or spectral characteristics lend themselves to various fields of applications, such as metrology, pulse compression, or spectroscopy. By providing some unique features, integrated SC sources can be particularly relevant to them.

### 7.1 Metrology

One of the first applications of coherent, broadband SCs was the self-referencing of frequency combs, which requires measuring *f*_CEO_. In fact, SCG in optical fibers was a prerequisite for a practical implementation of the common self-referencing via e.g. the f-to-2f scheme, and therefore it played an important role for the development of frequency metrology via frequency combs which was also honored with a Nobel Prize [169, 170]. Not surprisingly, SCG in waveguides has been used for the self-referencing of frequency combs, too.

**Figure 17:**
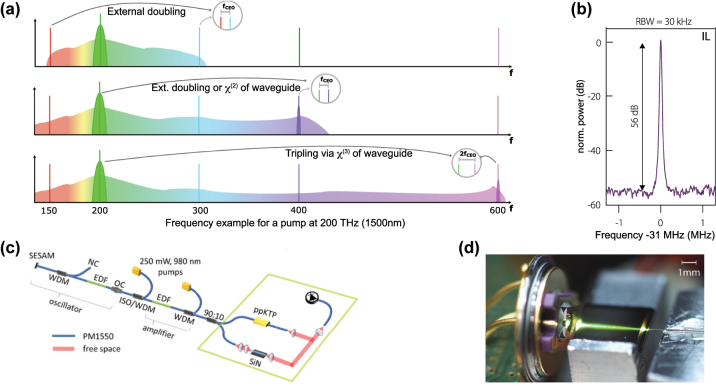
Nanophotonic waveguides for self-referencing applications. (a) A sketch showing different schemes to self-reference a mode-locked laser (for illustration purposes here centered at 200 THz) using SC and nonlinear multiplication. The top scheme is the one usually used when generating the SC in a fiber. The bottom two are mostly used in waveguides due to the very high efficiency of the SCG and the ability to efficiently double the pump. (b) Example of a *f*_CEO_ beatnote with a very high SNR detected right after an LNOI waveguide for a pump at around 1560 nm. (c) The full setup of a self-referenced fiber laser, here using a silicon nitride waveguide and external doubling in a periodically-poled KTP crystal (ppKTP). The components required for self-referencing are marked with a green box. (d) Thanks to the intrinsic *χ* ^(2)^ nonlinearity of LN, all components of the green box can be integrated into a volume of around 1 cm^3^. This configuration was used to generate the signal of (b). Both (c) and (d) use the center scheme of (a) in the two indicated variations for the doubling. Panels (b) and (d) adapted from [62], panel (c) adapted from [171].

To implement the f-to-2f scheme, a bandwidth of an octave and a frequency doubling of the red part of the spectrum are required (Figure 17(a), top). When beating the frequency doubled red part of the SC with its blue part, the offset frequency of the comb can be detected as a direct beatnote between the two components, because 2(*f*_CEO_ + *nf*_rep_) − (*f*_CEO_ + 2*nf*_rep_) = *f*_CEO_. Other schemes, such as 2f–3f and so on, require less optical bandwidth but higher order frequency multiplication afterwards, which becomes inefficient. Therefore, the large majority of conventional implementations relies on the f-to-2f scheme. However, due to the extremely efficient and broadband SCGs generated in waveguides, the situation here is a bit different. In the majority of cases, the bandwidth is larger than an octave, such that doubling the pump itself and beating it with the blue part of the SC spectrum is sufficient (Figure 17(a), center). In some waveguide platforms, even the f–3f scheme has been demonstrated, where the pump is tripled inside the waveguide via the Kerr nonlinearity (Figure 17(a), bottom).

**Figure 18:**
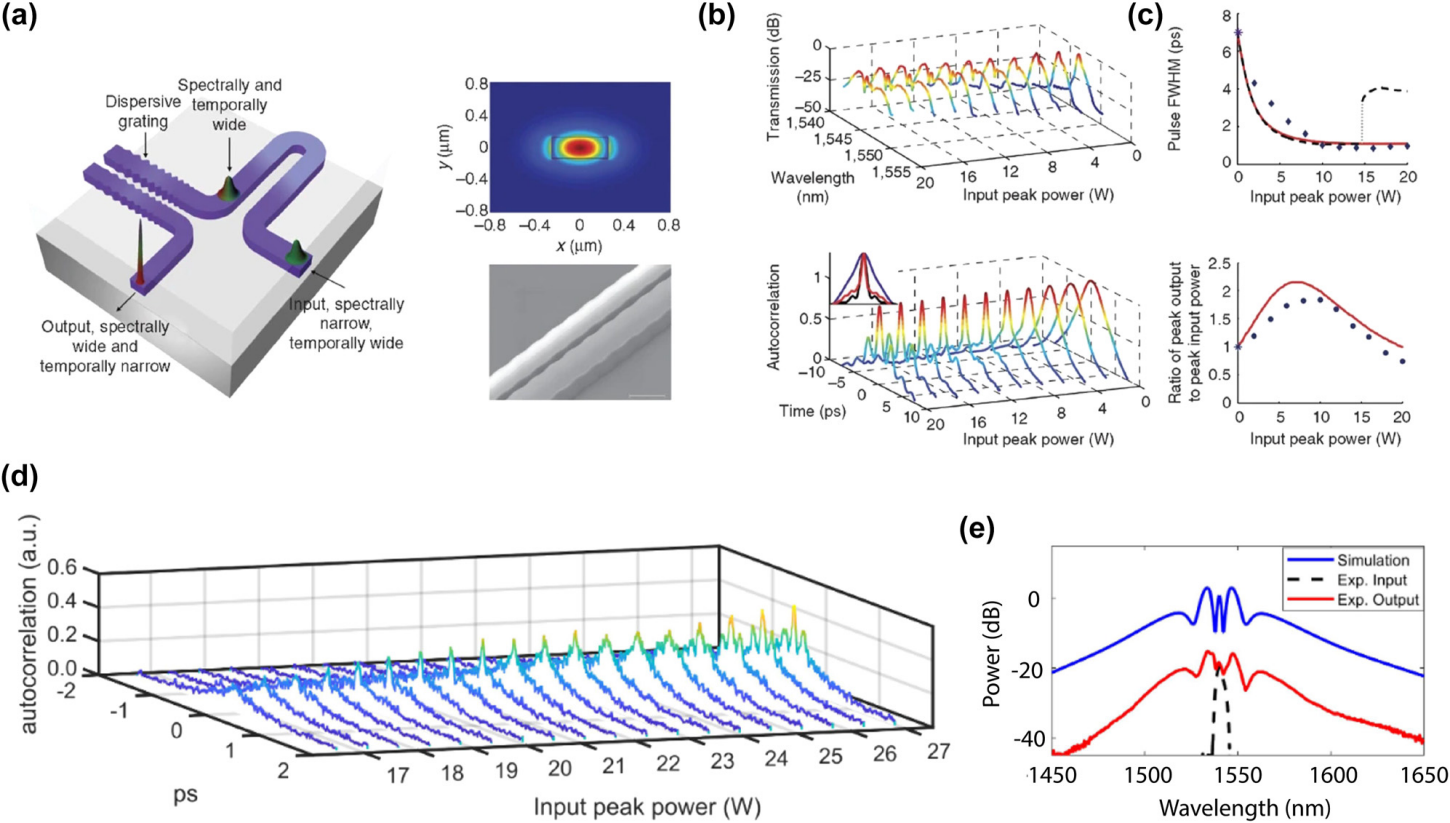
On-chip pulse compression. (a) Schematic of a two-stage pulse compression, calculated quasi-TE mode profile for silicon nanowire waveguide used for SPM (right, top) and SEM of dispersive grating (right, bottom). (b) Spectral output (top) and autocorrelation (bottom) as a function of input peak power. (c) Measured and calculated (red line) FWHM of the output pulse (top) and ratio of output peak power to input peak power, normalized to coupling and propagation losses as a function of input peak power. (d) Sequence of autocorrelation traces at the output of a 40 cm SiN waveguide while varying the input pulse peak power. (e) Experimental input and output spectra. Panels (a)–(c) adapted from [172]. Panels (d) and (e) adapted from [173].

Compared to other applications of SC spectra, there are a few distinct characteristics of SCG that are specifically relevant to this application. If the SC is only generated for self-referencing via the f-to-2f scheme, only the power at f and 2f components is important. The power of the spectrum in between can be very low or highly modulated without causing problems. Similarly, any bandwidth beyond the octave is not useful. Therefore, effects like DWs and Raman can be exploited to obtain an increase of power at the desired frequencies. Furthermore, several demonstrations have not only shown SCG inside the waveguide but have obtained the required frequency doubling or tripling inside the waveguide as well (Figure 17(d)), which is a substantial advantage over fiber-based SCG where this frequency doubling is usually implemented in a dedicate and subsequent stage, often leveraging free-space optics.

Silicon nitride is one of the most mature integrated platforms. As such, it has been exploited within several demonstrations of self-referencing. The first experiments only showed a bandwidth of an octave, sufficient for self-referencing with external doubling as in the scheme of Figure 17(a), top [78]. Later experiments showed also f–3f, where the frequency tripling via the Kerr nonlinearity of Si_3_N_4_ leads to the detection of 2*f*_CEO_ at usually triple the pump frequency (e.g. near 520 nm in the first demonstration with a pump laser in the telecom range) [174, 175]. Later work on Si_3_N_4_ made use of an optical poling effect [176, 177], that had been observed to introduce an effective second-order nonlinearity in Si_3_N_4_ [178]. This allows for somewhat efficient f-to-2f implementations also without any external doubling. With Si_3_N_4_, the SNR for the detection of *f*_CEO_ can reach above 30 dB. In a different demonstration, the SC from a Si_3_N_4_ waveguide was used to stabilize a mode-locked laser to two optical reference lasers [179]. Such configurations can be used to generate ultra-stable RF frequencies or as a part of an optical atomic lock. The potential of using integrated optics, again with a Si_3_N_4_ waveguide, instead of fiber optics for self-referencing was nicely demonstrated by a full, self-referenced laser system which draws less than 5W of power as illustrated in Figure 17(c) [171]. The progress in this technology is very fast, and optical components relying on integrated photonics for efficient self-referencing, are by now commercially available [180].

Direct doubling in the SCG waveguide can be also implemented using material platforms with intrinsic second-order nonlinearity, such as LN and AlN. Demonstrations with these platforms have indeed shown the potential for very compact and power efficient, yet high-performance self-referencing stages. First demonstrations in AlN [35] and LN [128, 129, 134, 181] showed SNRs for the electric self-referencing signal of already 37 dB and up to 39 dB, respectively. Recent demonstrations using regular LNOI waveguides on the other hand even reached SNR levels above 55 dB using standard photodiodes [62] (Figure 17(b) and (d)), which is on par with some of the best systems built from bulk components.

### 7.2 Pulse compression

Although temporal narrowing rather than spectral broadening is the main goal of pulse compression, it is intimately linked to SCG. In fact, both processes require precise control of nonlinearity and dispersion. Moreover, recent advances in the miniaturization of mode-locked lasers [182], as will be further discussed in Section 8, suggest the capability to build a chip-scale electrically driven SC source in the near future. However, such lasers still emit pulses longer than 1 ps so that pulse compression will therefore be of paramount importance for triggering subsequent SCG on-chip, as the latter mostly require sub-ps pump pulses. Whether it be to drive higher-order soliton dynamics in the anomalous dispersion regime, or to enable broadband SC in the SPM driven normal dispersion one, shorter pulses can indeed more readily trigger SCG than longer pulses. More generally, shorter pulses can deliver higher peak power and can maintain the coherence of SCG. While on the one hand, pulse compression represents a nice functionality for developing fully integrated on-chip systems in the long run, on the other hand, spectral broadening mechanism underlying SCG, such as high order soliton dynamics and SPM, are involved in the compression of optical pulses, as will be discussed below. Finally, beyond its use for and link with SCG, pulse compression finds applications in many fields like metrology [170, 183], biology [184], optical communication [185] and high harmonic generation toward UV or soft X-ray frequency [186, 187], and to study physical mechanisms at the attosecond time-scale.

Traditionally, pulse compression mechanisms include two stages: a first one where the spectrum is broadened by optical nonlinear effects, and a second one in which the pulse is re-compressed. Usually, SPM in nonlinear bulk crystals, HNLFs, or PCFs with normal dispersion are used in the first stage as one can obtain linear frequency chirp. This can be compensated in the second stage by different optical components such as diffraction gratings, prism pairs, chirped mirrors, or Bragg gratings. As a result of spectral broadening and chirp compensation, the pulse duration is reduced after the second stage. Although leading to good quality pulses with low pedestals, the pulse temporal stretching in the first stage decreases the pulse peak power, reducing consequently the nonlinear phase shift and spectral broadening and therefore the temporal compression which can be obtained. Another technique relies on high-order soliton dynamics to compress optical pulses in a single stage. When a pulse is injected in a nonlinear waveguide with *L*_D_ > *L*_NL_, it propagates in a high soliton state and undergoes a periodic evolution, exhibiting local temporal compression, proportional to the soliton number. By appropriately choosing the power, dispersion and length parameters, the pulse can reach its minimum temporal duration at the end of the waveguide. With this technique higher compression factors can be achieved, at the expenses of a pedestal in the temporal profile of the pulse, due to the uncompensated nonlinear frequency chirp. Finally, self-similar compression has been investigated theoretically and experimentally in optical fibers [188–191]. In this case, temporal compression can be achieved employing varying nonlinearity and/or dispersion profiles along the propagation direction of the pulse. The possibility to precisely engineer, along the propagation direction, the dispersion and the nonlinear parameters of integrated photonic waveguides is a valuable tool for bringing the different compression schemes to a chip-scale device operating at low power levels. A comprehensive review of on-chip pulse compression can be found in [192].

A first demonstration of an on-chip pulse compressor was reported by Tan et al. [172] using a dual stage scheme (Figure 18(a)–(c)). The authors achieved a compression factor as high as 7, resulting in final pulse duration of about 1 ps, in a 6 mm long device. In the first stage, SPM was generated in an SOI waveguide and a subsequent on chip Bragg grating was implemented as a dispersive element for the second stage. More recently, a compression factor of 11, leading to 550 fs compressed pulse duration, has been demonstrated in a similar design by using a 5.5 mm long ultra-silicon-rich nitride on insulator waveguide [193]. The most investigated on-chip pulse compression is based on high order soliton dynamics. One of the first demonstrations achieved sub-picosecond pulse duration in a 45 mm long Hydex waveguide [194] with a compression factor of 2. Such approach recently led to much higher compression factors [173, 195] (Figure 18(d) and (e)) and pulses in the few cycle regimes [196].

An interesting advantage of nanophotonic waveguides is that they can be precisely varied or structured along the direction of the pulse propagation. For instance, waveguide tapers have been proposed for self-similar pulse compression [190, 191]. Also, waveguide-based Bragg gratings and photonic crystals can dramatically increase the nonlinear interaction via slow-light phenomena, and offer an enhanced dispersion variation close to the photonic band-edge. These properties have been exploited to reduce the length of the pulse compressor in the sub-millimeter scale and the energy of the required input pulses (down to the 10 pJ level) within a single slow-light engineered photonic crystal waveguide [36, 197], [198], [199].

### 7.3 Spectroscopy

SC sources can provide spectra with relatively large power spectral densities combined with ultrahigh bandwidth. Such spectra appear as great candidates for spectroscopic applications, especially when the bandwidth is pushed further towards longer wavelengths as to enter the field of MIR spectroscopy and sensing. For many molecules, the MIR spectral region contains strong absorption lines, induced by various vibrational modes, resulting in an optical absorption fingerprint for these molecules. One way to measure this fingerprint is to record spectra of the interaction of the molecules of interest with a broadband MIR light. The analysis of the measured spectra allows for the detection, identification, and quantification of a variety of molecules. While direct MIR light generation is possible with, for example, quantum cascade lasers (QCLs) and interband cascade lasers, nonlinear wavelength conversion has always been considered as a particularly interesting option to generate MIR light as it has the potential to cover parts of the spectrum not easily reachable with other means. Established ways include optical parametric oscillators (OPOs) and difference-frequency generation between two strong continuous wave lasers to provide efficient conversion towards the MIR. In contrast to these approaches, SCG can provide broader and tunable emission, with the added benefit of a single pass geometric without the need for either additional seed or temporal synchronization.

SC sources based on optical fibers have already attracted a lot of attention for spectroscopy applications, as confirmed by a large body of work [200], and integrated SC is also showing great potential in that respect. Various platforms have shown potential for direct absorption spectroscopy. In [118], a ChG waveguide was used as to combine both SCG and chemical detection by evanescent field sensing. From a compact 1560 nm femtosecond pump source, the SC is generated in an unclad ChG with up to 21 mm in length. For a waveguide with a cross section of 950 nm × 400 nm, the SC extended between 1380 and 2050 nm. By immersing the waveguide in chloroform solutions, the overtone absorption peak centered at 1695 nm was used to quantify the performance of the device, providing a proof-of-principle of such combined platform, albeit with limited performance at this point owing to the high loss of the waveguide and the weak evanescent field. The potential of MIR SCG in SiGe waveguides for direct absorption spectroscopy was also demonstrated with a proof-of-principle experiment [88]. Using 2.5–5 μm light directly emitted by SCG in dispersion engineered SiGe waveguides, the absorption fingerprint of both water and CO_2_ could be detected and identified in parallel.

But the largest body of spectroscopy applications, not only for direct absorption but also dual-comb techniques, has been carried in Si and Si_3_N_4_ waveguides. Nader et al. demonstrated in [73] dual-comb spectroscopy at 5 μm. They generated two coherently broadened spectra from a pump at 3.06 μm (obtained from DFG in a periodically poled lithium niobate crystal – PPLN): one in an SOS waveguide and another one directly from a PPLN. As the pump source of the dual-comb system originated from DFG, the two MIR combs were offset free such that only the stabilization of the repetition rate was required. The two combs were locked and stabilized with a slight Δ*f* difference in repetition rate using a microwave circuit. The dual-comb system was then benchmarked to detect carbonyl sulfide with ro-vibrational lines from 4.7 to 5 μm. Because of the sapphire absorption, which limits operation to 6 μm, a suspended SOI structure was used in [58] in order to push the operation deeper in the MIR. Similar to [73], one comb came from the suspended SOI waveguide pumped at 3.06 μm while the second was generated from intrapulse DFG in an orientation patterned GaP (OP-GaP) bulk crystal. The dual-comb spectroscopy setup was used for gas and liquid phase sensing: using various waveguide dimensions as to control the generation of the DW, the measurements could be carried out between 4.9 and 8.8 μm with 100 MHz resolution and mW level average power, hence obtaining performance equivalent to other, yet bulkier MIR dual-comb systems.

**Figure 19:**
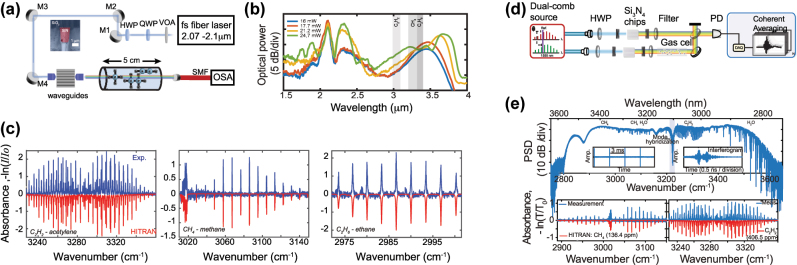
Spectroscopy applications of on-chip SCG. (a) Experimental setup for absorption spectroscopy. (b) Experimental SCG generation in a SiN waveguide pumped at 2.1 μm and different pump powers. (c) Normalized experimental gas absorbance (blue) and the HITRAN database (red) for C_2_H_2_, CH_4_, and C_2_H_6_. (d) Schematic of dual-comb setup where the two combs are generated in SiN waveguides. (e) Retrieved MIR spectrum from the detected and coherent averaged (52 s) interferogram trace. (g) Measured gas absorbance (blue), CH_4_ (left) and C_2_H_2_ (right), fitted and compared with the HITRAN database. Panels (a)–(c) adapted from [201], panels (d) and (e) adapted from [202].

Several proof-of-principle demonstrations using Si_3_N_4_ waveguides also established their high potential for such application. The efficient generation of a 3.05 μm DW in a large cross-section Si_3_N_4_ waveguide pumped by a 2 μm fiber laser was leveraged for MIR absorption spectroscopy of acetylene [82]. The light from the Si_3_N_4_ waveguide was directly coupled into a gas cell and the transmitted spectrum was collected by an optical spectrum analyzer where clear dips showing an excellent agreement with the HITRAN database were measured. In [201], this source was further optimized by controlling the pump wavelength and the waveguide geometry. The spectral coverage of a single DW could be significantly enhanced, reaching a 10 dB bandwidth of 1000 nm around 3.5 μm, without sacrificing efficiency. As such the MIR source from one Si_3_N_4_ waveguide was used for simultaneous and discrete detection of multiple gas-phase molecules in a single gas cell: acetylene, methane, and ethane were detected with a simple direct-absorption spectroscopy setup (Figure 19(a)–(c)). The coherence of a DW generated in Si_3_N_4_ was also exploited in a dual-comb experiment, where both combs were generated in integrated waveguides, the dispersion of which was engineered based on a coupled structure to flatten and extend the integrated dispersion on the long wavelength side [202]. The two waveguides were pumped by two ultralow noise carrier-offset frequency locked femtosecond lasers at 1550 nm with a small repetition frequency difference of 320 Hz. As the used MIR part of the SC is the spectral extension of the original pump source obtained by soliton dynamics with a low soliton number (*N* around 5), it inherits the properties of the input source comb. The work demonstrated a phase-resolved MIR dual-comb spectrometer with a performance competitive to DFG-based dual-comb spectrometers (Figure 19(d) and (e)).

### 7.4 Other

Optical coherence tomography (OCT) is one of the most successful optical imaging techniques, thanks to its ability to provide real-time, non-invasive, label-free, and noncontact three-dimensional sample visualization. The axial resolution of OCT systems is proportional to the square of the central wavelength of the light source, and inversely proportional to its bandwidth [203]. Using short wavelength sources improves the resolution, but it might reduce the penetration length due to absorption and scattering of light in some types of samples, such as composite paint samples, intralipids, rubber, and ceramic materials [204]. A more generally valid approach to improve the resolution is to use a broadband source, such as a SC. Fiber–based SC sources are now widely used in commercially available OCT systems [203]. The main disadvantage of SC sources for OCT is the high intensity noise and pulse-to-pulse fluctuations that are typical of SC generation in the anomalous dispersion regime (the common regime for commercial SC sources), which limits the sensitivity of the system. Recently, it has been shown that using a low noise ANDi fiber-based SC source improves the contrast, sensitivity and penetration depth of the OCT system [203]. Regardless of the dispersion regime, optical fibers require high power to generate the SC, and the spectrum needs to be shaped and attenuated with bulk optical filters to be safely used for imaging. On the contrary, on-chip SC generation can be achieved at low input powers, thanks to the high mode confinement in the waveguide core. Recently, Ji and coworkers reported the first OCT system using a millimeter-scale chip-based SC source [205]. The authors harnessed a low noise spectrum generated mainly by SPM in a Si_3_N_4_ chip with pump pulse energy of 25 pJ. As compared to systems based on a commercial fiber-based SC source, they demonstrated higher sensitivity without the need for any post filtering. Integrated SC sources are therefore promising for compact and high performance OCT systems. The use of SC sources for imaging is not limited to OCT, and fiber-based sources have been successfully employed for MIR reflectance microspectroscopy [206], coherent anti-Stokes Raman scattering microscopy [207], chromatic confocal microscopy [208], spectroscopic photoacoustic imaging [209], and various other imaging techniques [210].

Another area of application is telecommunication. There is an undeniable and evident growth in traffic demand, pushing to the limit the bandwidth of optical systems. Current commercial systems rely on wavelength division multiplexing (WDM), polarization-division multiplexing (PDM), while space division multiplexing exploiting multi-core or multi-mode fibers is an active area of research. Regardless of the approach, massive parallelization is required, such that arrays of discrete wavelength sources become an essential element to optimize, in particular to reduce energy consumption and size. Optical frequency combs therefore appear as an excellent alternative to common laser arrays, with each comb line replacing one individual laser. Coherent SCG, which can achieve large spectral broadening with good conversion efficiency while being more robust, is an interesting chip-based alternative to approaches that make use of either integrated mode-locked lasers [211] or soliton Kerr comb generation in integrated microresonators [212–214]. The SCG approach was initially validated using spectral broadening in highly nonlinear fibers of an initially narrow frequency comb with GHz repetition rate [215]. A difficulty for the integrated approach comes from the fact that most nanophotonic SC sources rely on the use of pulses with high peak power, owing to the short waveguide lengths and high dispersion. The typically used femtosecond pulse trains with low repetition rates (<GHz) do not satisfy the requirements for telecommunication as the pulse repetition rate determines the frequency spacing of the possible WDM channels. Given that the rate should be in the tens of GHz, this leads to a much reduced peak to average power ratio of the pump pulses and as a result efficient broadening is challenging to reach. Solutions lie in the use of a platform with a very high nonlinearity, or with low and flat dispersion together with low loss as to enable long propagation distances. By leveraging the very high nonlinearity of AlGaAs on insulator, the authors in [138] showed the broadening of a 10 GHz picosecond pulse in a 5 mm long waveguide. The frequency comb was broadened to efficiently cover the entire telecom band while maintaining a narrow linewidth on the extended comb lines. The source was then investigated as a potential multichannel array in a multidimensional modulation and multiplexing scheme, proving that it could sustain several hundreds of Tb/s and potentially replace hundreds of parallel lasers [138]. The potential of long and low dispersion waveguides was also recently validated for the controlled broadening of GHz pulses. In [81], the authors relied on a 20 cm long spiral Si_3_N_4_ waveguide with low ANDi to coherently broaden a spectrum centered at 1550 nm. A span from 1525 to 1575 nm could be generated from a 0.5 ps pump pulse with a 25 GHz repetition rate, initially generated from simple electro-optic modulation. The generated comb has however not yet been used as a source for WDM systems.

After this review of the various demonstrations that showed how SCG in chip-based devices could be efficiently leveraged within diverse application areas, we end by discussing next some prospects for this emerging technology and the related challenges that still need to be addressed.

## 8 Perspectives and conclusions

The work done on the generation of SC in integrated photonic platforms is, as evident from this Review, plentiful with many already groundbreaking demonstrations. However, the road towards end-users of these integrated systems is still very long. Fiber-based SC sources are, on the other hand, already commercially available. So what are the advantages and perspectives for integrated SCG, as to take this approach from a scientific (laboratory) curiosity to the market?

**Figure 20:**
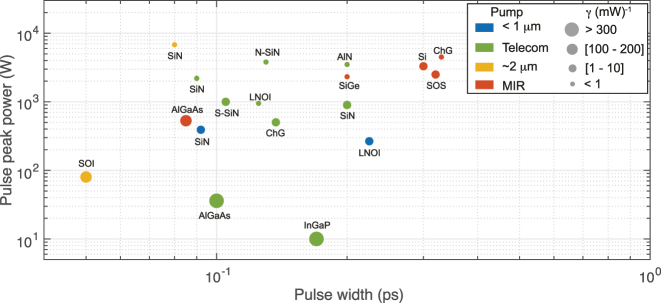
Peak power and pulse width for various demonstrations of octave spanning SC in chip-based platforms. The colors indicate the wavelength of the pump while the size of the marker indicates the magnitude of the nonlinear coefficient *γ* of the used platform.

A significant limiting factor is the required pump source. Even though the broadening waveguiding medium is integrated, and requires only very short propagation length in the mm range, the benefit of size, weight, cost, and energy reduction is not there yet. This is because all work is still being conducted with expensive and specialty short pulse laser sources, even in the platforms with the highest nonlinearities. To move the technology forward, it is probably necessary to think as the pump source and the broadening section as a whole, with a global goal of being compact, portable, and cost effective. In the last few years, we have seen some steps taken in that direction, with the demonstrations of telecom fiber-laser driven extremely broad SCG. However, as evident from Figure 20, where the pump power and pulse width used for octave spanning demonstrations in chip-based platforms are plotted, we are quite far from electrically driven SCG. Indeed, lifting the constraints on both the peak power and pulse duration for generating SC out of these platforms, so as to get closer to the capabilities of compact semiconductor pulsed lasers, appears quite difficult to reach from the current state-of-the-art. One trend is obvious though: in order to lower the pump requirements (and move towards the lower right hand corner of the graph), materials with high values of *γ* (represented as bigger dots on Figure 20) are necessary. While an electrically driven SCG might therefore seem, at this moment, difficult to envision, the rapid progress in several other key domains gives a new impulse for fulfilling this goal. First, there is constant progress in integration, pushing the development of monolithic III–V and heterogeneous III–V-on-silicon on chip mode-locked lasers [216, 217]. On chip passive extended waveguide cavities, both in III–V or silicon, can also be used to realize low-noise, narrow-linewidth mode-locked lasers [218, 219]. With such extended cavities, the pulse repetition rate can, at the moment, reach down to the GHz [220]. When leveraging Si_3_N_4_ low linear and nonlinear losses, and small thermo-optic coefficient, the pulse energy can be scaled up. In [182] a III–V-on-SiN electrically pumped mode-locked laser emitting at 1600 nm with a repetition rate of 3 GHz was demonstrated, having an expected transform-limited pulse width of 1.4 ps, and an on-chip pulse energy of 2 pJ (corresponding to 1.4 W peak power). While such characteristics already satisfy resonant SCG [221], the peak powers of such sources are still too low considering the typical kW peak power required (see Figure 20). Another way of generating on-chip pulsed sources without mode-locking is by using integrated electro-optic (EO) modulation. Sub-picosecond pulses from a CW single-frequency laser are obtained by a cascade of EO modulators, in a robust and frequency-agile method. However, in such table-top systems, many discrete EO modulators and RF amplifiers are required, due to the relatively high V_*π*_ of the modulators. Chip-scale versions are becoming popular, driven by the fast progress on LNOI [222]. Very recent works reported 520 fs pulse source at 30 GHz with 0.54 pJ pulse energy formed by a full on-chip time-lens system, using cascaded low-loss EO amplitude and phase modulators and chirped Bragg grating [223], whereas a 336 fs pulse source was demonstrated with a 30.925 GHz repetition rate and about 1 W peak from a mutually coupled resonators structure [224]. Again, the peak powers remain rather low for SCG such that amplification would be needed, which at the moment is still done off-chip with table-top fiber doped amplifiers, when operating at such telecom wavelength. High power integrated erbium (Er) amplifiers seem to be a missing link, with their realization particularly challenging owing to constraints in gain from achievable doping concentration. There have been demonstrations of Er doped waveguide amplifiers (EDWA) with net gain, using for example Er doped Al_2_O_3_ [225–227], or TeO_2_ [228, 229], but the achievable output powers are below what can be achieved with heterogeneous integration [230] and far from the levels necessary for SCG. Recently, a 30 dB small signal gain, with 145 mW of output power, was reported in an integrated erbium-implanted Si_3_N_4_ 1 m long waveguide [231]. The high performance of the device comes from the large overlap between the embedded ions, obtained through three successive implantation steps directly at the wafer-scale, and the optical waveguide mode.

Beyond the integration of the SCG section together with the pump source, we can look at other added functionalities, which can also be directly implemented at the chip scale, and might help towards the realization of efficient and fully integrated SC sources. It is indeed possible to engineer different sections of the device as to carry specific tasks, for example by cascading sections of waveguides with different dispersion properties for extending and/or shaping the output SC, as covered in previous chapters. As presented in Section 7, one could, for instance, consider pulse compression as a desirable additional building block of a complete SCG system. Indeed, combining pulse compression, ideally with amplification, so as to feed a nonlinear waveguide stage could be a way towards fully integrated octave spanning SCG. As we previously discussed, pulse compression can be achieved both in standard waveguides and in highly compact PhC waveguides. The interest in PhC waveguides as a building block of an on-chip SCG system is not limited to temporal pulse compression. The ability to strongly engineer the dispersion of PhC waveguides enables to spatially compress the pulse in the so called slow-light regime. Thanks to the increased light–matter interaction, slow-light can significantly enhance nonlinear effects, thereby reducing the pump peak power requirements [232, 233]. While the fundamental limit in the delay-bandwidth product [232] afforded by PhC waveguides, however, makes slow-light an impractical way to directly enhance the efficiency of ultra-broadband effects such as SCG, they can locally boost nonlinear processes and be effectively interfaced with and be helpful to other (broadband) integrated nonlinear devices. For instance, PhC waveguides have been used for the miniaturization of optical parametric oscillators (OPO) [234]. The difficulty of integrating pulsed MIR lasers for the generation of MIR SC could be overcome by using an integrated OPO as an additional building block of the SCG system, although integrated OPOs reaching the MIR are yet to be demonstrated. Besides the integration of several nonlinear functions, the heterogeneous integration of materials with spectrally distinct linear and nonlinear properties could be an interesting alternative for the generation of an MIR SC without the requirement of MIR pump sources. Cascaded sections of different materials could be employed to first generate an SC at shorter wavelengths from a near-IR pump, and then extend the spectrum towards longer wavelengths in the subsequent sections. This approach has been successfully employed for the generation of an MIR SC in a cascaded fiber system [235].

While dispersion engineering, either of simple single waveguide structures or more complex cascaded/coupled ones, is a powerful way to shape and tailor the SC, more fine control of the nonlinear dynamics are sometimes required. To that end, it is possible to use machine learning methods in order to have some additional control of the coherent dynamics. For example, genetic algorithms were used to prepare custom pulse-trains with an integrated pulse-splitter as to maximise the spectral density in defined wavelength ranges of the SC [236, 237]. It was also shown that Gaussian-like peaks can be intentionally positioned in the spectrum of the generated SC, by changing the spectral phase of the incoming pulse with a genetic algorithm [238]. While at the moment all the work using maching learning to tailor SCG was carried out for systems using optical fibers, it should be possible to apply the same approaches to integrated systems and further push the performance and applications of these sources.

**Figure 21:**
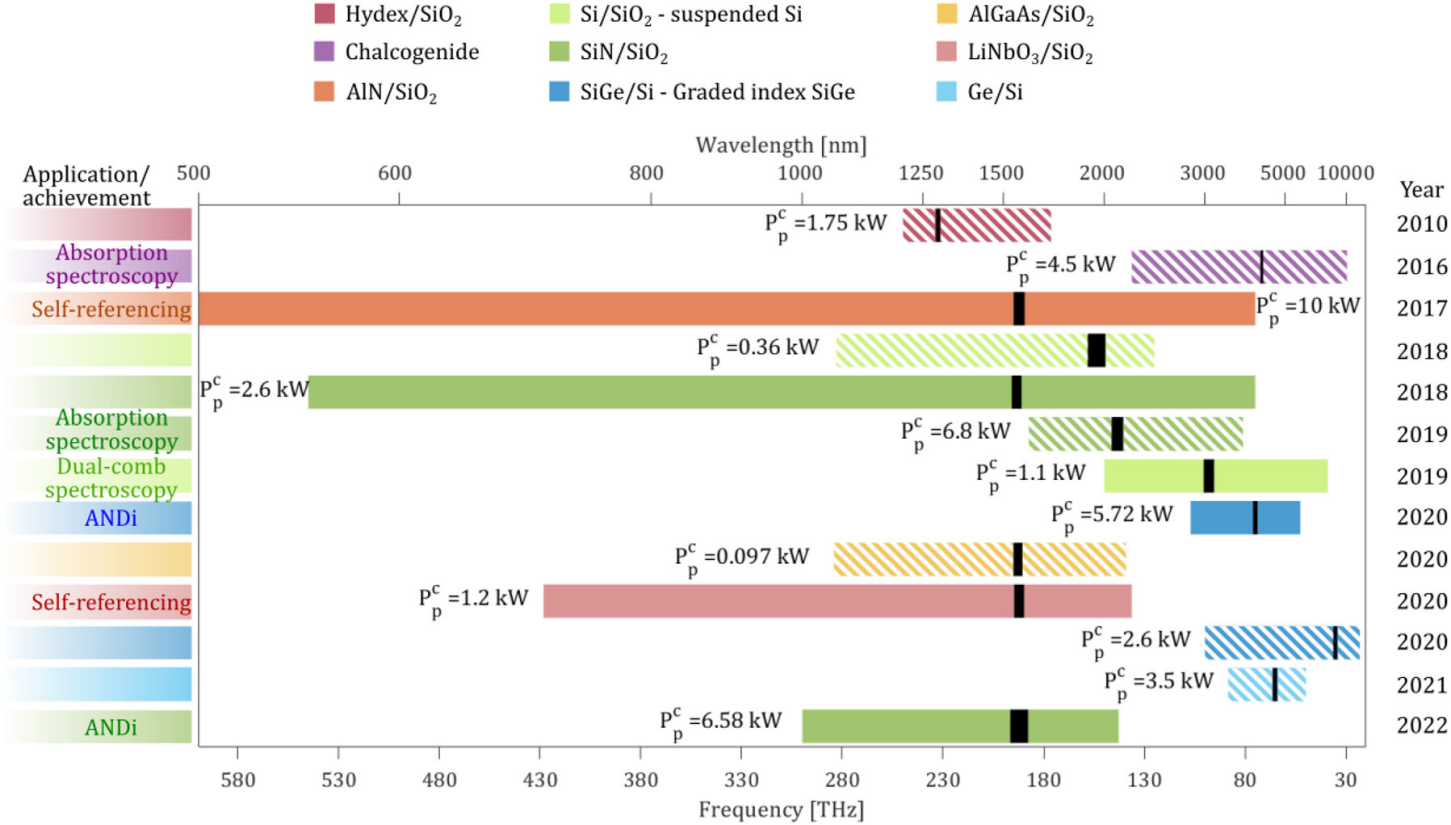
Comparison of selected demonstrations of SCG in integrated platforms (Refs. from top to bottom of the figure: [15, 25, 28, 35, 55–60, 81, 82, 129]). For each result, the width of the colored band indicates the bandwidth of the generated SC, the color indicates the type of platform, and $P_{\text{P}}^{\text{C}}$ the coupled peak power. Unpatterned bands indicate coherent SCG. The black lines represent the pump wavelength/frequency. The bandwidth of the pump was inferred from the pump pulse duration considering transform limited Gaussian pulses.

In conclusion, since the first demonstrations at the beginning of the years 2010s, on-chip SCG has seen a fast growth, fuelled by the development in the fabrication of photonic integrated waveguides, and is now starting to quit scientific laboratories and become commercially available, although with off-chip pump sources at the moment. As we showed in this Review, SCG has been demonstrated in a number of integrated platforms, each with its own strengths and weaknesses. The number of meaningful properties (e.g. spectral bandwidth, power, and coherence), the importance of one property or the other depending on the envisioned application, as well as the different experimental conditions in each demonstration (e.g. pump pulses duration and repetition rate), make the comparison between different platforms non trivial. In Figure 21 we try to highlight the main features of selected SCG demonstrations in the most common and developed platforms. For SCG, the most obvious thing is to compare the bandwidth, which in the figure is represented by the width of the colored bands. However, as we mentioned in Section 2, the definition of SC bandwidth in different publications is not univocal. Most often, the −30 dB level is considered, but it is not unusual to define it at the −20 or −40 dB level, or even just the spectral region in which the signal rises above the noise floor of the spectrometer. In addition, SCG in the anomalous dispersion regime often results in uneven and modulated spectra, and wide low-signal spectral regions might be present between the central part of the spectrum and the DW or SH at the extremes. Again, the importance of having a flat spectrum strongly depends on the envisioned application. Despite everything, it is evident from the figure that each platform is well suited to cover a certain wavelength range, mostly due to the transparency of the materials. Phase coherence is also an important feature for applications like dual-comb spectroscopy. In the figure, we represented coherent SCs with unpatterned bands. We considered an SC to be coherent when it was experimentally demonstrated or inferred through self-referencing or dual–comb spectroscopy, or when no decoherence mechanism is involved in the SCG process, like in the ANDi regime. Finally, the required coupled peak power $P_{\text{P}}^{\text{C}}$ is indicated next to each demonstration. The power tends to be higher in the MIR because larger cross-sections are needed to accommodate MIR light, leading to a lower nonlinear coefficient. As we tried to highlight throughout this Review, the single ideal platform does not exist, at least not yet. However, the large choice of platforms, along with the possibility to tailor the dispersion regime of operation as well as, in the long term, to heterogeneously integrate them, offers ample opportunities to match the needs of a given application.
