# Erratum to: Supercontinuum in integrated photonics: generation, applications, challenges and perspectives

**DOI:** 10.1515/nanoph-2023-0405

**Published:** 2023-07-20

**Authors:** Camille-Sophie Brès, Alberto Della Torre, Davide Grassani, Victor Brasch, Christian Grillet, Christelle Monat

**Affiliations:** Photonic Systems Laboratory (PHOSL), Ecole Polytechnique Fédérale de Lausanne, 1015 Lausanne, Switzerland; Université de Lyon, Institut des Nanotechnologies de Lyon (INL) UMR CNRS 5270, Ecole Centrale de Lyon, 69131 Ecully, France; Centre Suisse d’Electronique et de Microtechnique (CSEM), 2000 Neuchâtel, Switzerland; Q.ANT GmbH, 70565 Stuttgart, Germany

After the publication of the paper [1], the authors realized that the derivation of the nonlinear parameter *γ* is more appropriately written as:

(1) $$\gamma =\frac{3\chi^{\left(3\right)}\omega_{0}}{8n_{0}c}\frac{\underset{A\text{core}}{\iint }|F\left(x,y\right){|}^{4}\text{d}x\text{d}y}{\overset{\infty }{\underset{-\infty }{\iint }}|F\left(x,y\right){|}^{2}\text{d}x\text{d}y}$$

where the integral at the numerator runs just over the core area. This is because *χ* ^(3)^ refers only to the core material. In fact, in photonic integrated waveguides, we can assume that *χ* ^(3)^ of the core material is much larger than the one of the cladding, which therefore does not contribute to the nonlinear processes.

Also, the authors found an error in Equation (6), which provides the definition of the effective area of the waveguide’s mode. Taking into account the above-mentioned definition of *γ*, the correct expression is:

(2) $$A_{\text{eff}}=\frac{{\left(\overset{\infty }{\underset{-\infty }{\iint }}|F\left(x,y\right){|}^{2}\text{d}x\text{d}y\right)}^{2}}{\underset{A_{\text{core}}}{\iint }|F\left(x,y\right){|}^{4}\text{d}x\text{d}y}$$
